# Accurate Prediction of Ligand Affinities for a Proton-Dependent Oligopeptide Transporter

**DOI:** 10.1016/j.chembiol.2015.11.015

**Published:** 2016-02-18

**Authors:** Firdaus Samsudin, Joanne L. Parker, Mark S.P. Sansom, Simon Newstead, Philip W. Fowler

**Affiliations:** 1 Department of Biochemistry, University of Oxford, South Parks Road, Oxford OX1 3QU, UK

## Abstract

Membrane transporters are critical modulators of drug pharmacokinetics, efficacy, and safety. One example is the proton-dependent oligopeptide transporter PepT1, also known as SLC15A1, which is responsible for the uptake of the β-lactam antibiotics and various peptide-based prodrugs. In this study, we modeled the binding of various peptides to a bacterial homolog, PepT_St_, and evaluated a range of computational methods for predicting the free energy of binding. Our results show that a hybrid approach (endpoint methods to classify peptides into good and poor binders and a theoretically exact method for refinement) is able to accurately predict affinities, which we validated using proteoliposome transport assays. Applying the method to a homology model of PepT1 suggests that the approach requires a high-quality structure to be accurate. Our study provides a blueprint for extending these computational methodologies to other pharmaceutically important transporter families.

## Highlights

• A hierarchical computational approach determines ligand affinities to transporters
• Lysine-containing dipeptides proposed to bind vertically like a tripeptide
• Experimental structures are vital for the accurate prediction of affinities
• A model of prodrug interactions to human PepT1 is suggested

## Graphical Abstract

**Figure:**
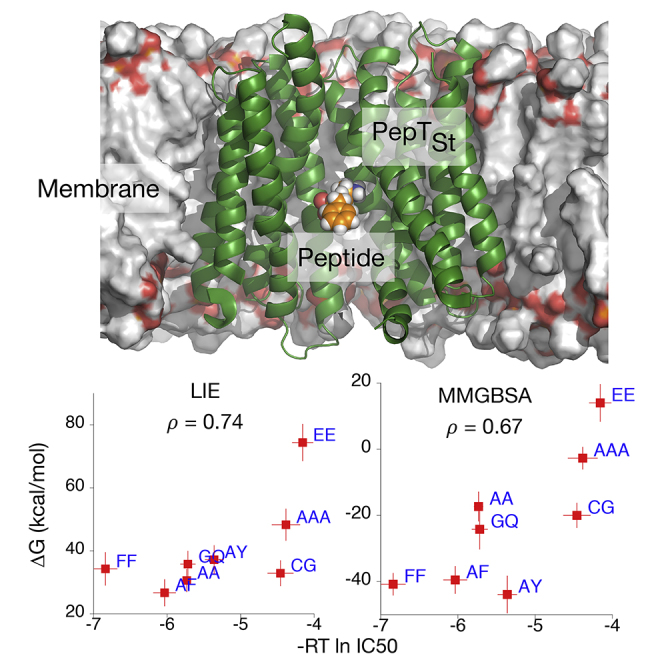

## Teaser

Samsudin et al. demonstrate how a hierarchical free energy method can predict the affinities of ligands for a peptide transporter protein. This approach may be used to optimize drug-transporter interactions.

## Introduction

The application of computational chemistry in drug development has centered for a long time around indirect ligand-based techniques, such as pharmacophore modeling and 3D-QSAR studies (Caporuscio and Tafi, 2011). Recently, the emergence of X-ray crystal structures of pharmaceutically important membrane proteins has shifted the paradigm toward direct structure-based approaches, for example, computing the free energy of binding of relevant ligands to a protein, and thereby finding or optimizing lead compounds. Scoring functions are currently the method of choice due to their cheap computational cost (Chen and Shoichet, 2009, Schlessinger et al., 2011, Geier et al., 2013). While this method can work for screening a large library of compounds to produce an initial list of candidates, more robust techniques are needed for accurately predicting binding, such as for ranking ligands, especially for highly dynamic proteins like membrane transporters.

Understanding how a drug candidate interacts with membrane transporters is becoming an important step in drug development (Giacomini et al., 2010). Compelling clinical evidence indicates that membrane transporters expressed in the epithelia of the intestine, kidney, and liver, and in the endothelia of the blood-brain barrier influence not only drug absorption and distribution (Dobson and Kell, 2008) but also their therapeutic efficacy and potential adverse reactions (Shitara and Sugiyama, 2002, Cusatis et al., 2006). A recent update of the U.S. Food and Drug Administration guidelines includes extensive recommendations on in vitro and in vivo studies of transporter-mediated drug-drug interactions (U.S. Department of Health and Human Services et al., 2012). Most of the key transporters that have been characterized belong to two major superfamilies: ATP-binding cassette transporters and solute carriers (SLCs). Of particular interest to this paper is the well-studied and pharmacologically important proton-dependent oligopeptide transporters (POT) family member, PepT1, also known as solute carrier family 15 member 1 (SLC15A1), which is the key representative of clinically important SLC transporters involved in drug transport.

PepT1 is expressed predominantly in the intestinal epithelia (Fei et al., 1994, Shen et al., 1999) and plays a crucial role in maintaining nitrogen homeostasis by coupling the uptake of dipeptides and tripeptides to the proton electrochemical gradient (Daniel and Spanier, 2006). Based on the 20 naturally occurring amino acids, there are more than 8,000 possible peptides that could be its substrates, most of which are expected to be transported (Ito et al., 2013). PepT1, therefore, has a highly promiscuous binding site that can accommodate a wide range of ligands with diverse structures and chemistries. In addition to nutritional peptides, it is well established that PepT1 also recognizes and transports a range of drug compounds, such as many β-lactam antibiotics (Luckner and Brandsch, 2005) and the tumor suppressor bestatin (Inui et al., 1992). The promiscuity of this transporter has been exploited in the development of prodrugs such as the antiviral acyclovir (Ganapathy et al., 1998) and the anti-hypotensive drug midodrine (Tsuda et al., 2006). In both cases, an amino acid residue was attached to the active drug moiety via an esterification reaction, leading to a compound that is transported across the lining of the gut by PepT1, thereby increasing its oral bioavailability. Understanding how ligands interact with these transporters and being able to predict their affinity could, therefore, enable the rational design of drugs with better pharmacokinetics (Brandsch et al., 2008).

Structurally, PepT1 belongs to the major facilitator superfamily (Pao et al., 1998) and so consists of 12 core transmembrane helices divided into N- and C-terminal bundles, with two additional helices observed in the bacterial homologs (Newstead, 2011, Solcan et al., 2012, Doki et al., 2013, Guettou et al., 2013, Zhao et al., 2014, Boggavarapu et al., 2015). The binding site is positioned between the two bundles and is exposed to either side of the membrane as per the alternating access mechanism (Radestock and Forrest, 2011), with distinct helices controlling this process in a cooperative scissor-like motion (Fowler et al., 2015). The bacterial isoforms share a high sequence identity with mammalian PepT1 within the peptide-binding site, suggesting there may be universally conserved binding and transport mechanisms (Newstead, 2014). Recent high-resolution structures of a homolog from *Streptococcus thermophilus*, PepT_St_, demonstrated that there are at least two binding orientations within this cavity: the dipeptide AlaPhe (PDB: 4D2C) adopted a horizontal pose with respect to the plane of the membrane, whereas the tripeptide AlaAlaAla (triAla, PDB: 4D2D) bound vertically (Lyons et al., 2014), consistent with the dual proton:peptide stoichiometry observed for this transporter (Parker et al., 2014). A second, lower-resolution study resolved the structures of two tripepides (AlaAlaAla, PDB: 4TPJ, and brominated AlaTyrAla, PDB: 4TPG) and a dipeptide (brominated AlaTyr, PDB: 4TPH) when bound to PepT_So2_ (Guettou et al., 2014). For this protein, all three peptides bound in the same horizontal pose with respect to the membrane. These studies have provided essential insights into the molecular basis of promiscuity by the POT transporters, but also raise a further question of how the numerous other di- and tripeptides and, more importantly, drugs interact?

In this article, we develop a computational approach for accurately predicting the affinities of ligands to the peptide transporters. We modeled the binding of various dipeptides based on the crystal structure of the PepT_St_-AlaPhe complex, and used a range of in silico free energy methods to predict their binding affinities. All these methods are very well established and have been applied to a wide range of protein-ligand systems (Chodera et al., 2011). We find that the moderately cheap endpoint methods provide a fast way to classify these ligands into good and poor binders. Our results suggest PepT_St_ generally prefers neutral over charged substrates. Applying a more rigorous theoretical method to a series of dipeptides reveals the importance of the N-terminal side chain in determining the overall affinity. Using a proton-driven competition uptake assay, we validated these predictions but found discrepancies with basic dipeptides. We suggest that these dipeptides bind in a vertical orientation, similar to the previously observed triAla PepT_St_ complex, to accommodate their large side chains. Applying the method to a homology model of human PepT1 suggests that the accuracy of this method depends on the quality of the available protein structure. Nevertheless, our results help to explain how the prodrug approach has worked for PepT1 by revealing the importance of the N- and C-terminal interactions in the binding site. Overall, this study demonstrates how in silico methodologies can work in tandem with in vitro assays to predict ligand affinities in a pharmaceutically relevant membrane transporter.

## Results

### Endpoint and Exact Free Energy Methods Accurately Predict Affinities

**Figure 1:**
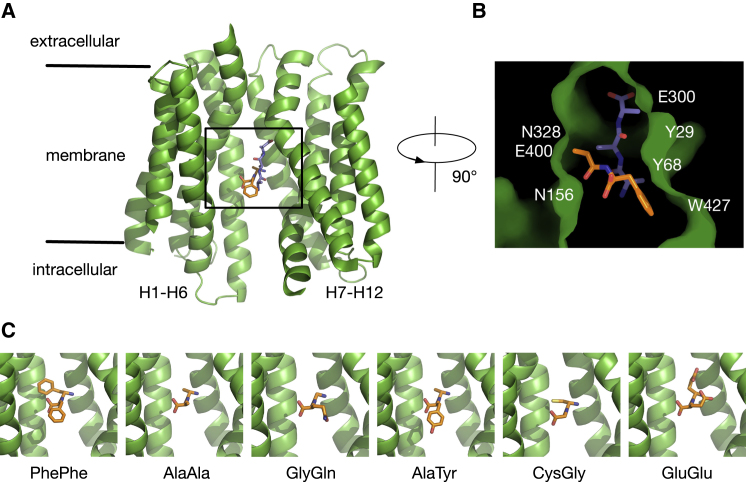
Modeling the Binding of Peptides Based on the Crystal Complexes of PepT_St_ (A) Superposition of the two binding modes of peptides to PepT_St_. The dipeptide AlaPhe (orange) binds in a horizontal orientation (PDB: 4D2C), whereas the tripeptide triAla (purple) binds in a vertical orientation (PDB: 4D2D) (Lyons et al., 2014). For clarity, transmembrane helices H2, H11, HA, and HB are removed. (B) Surface representation of the binding pocket of PepT_St_ with the positions of key interacting residues outlined. (C) Models of six dipeptides bound to PepT_St_.

**Figure 2:**
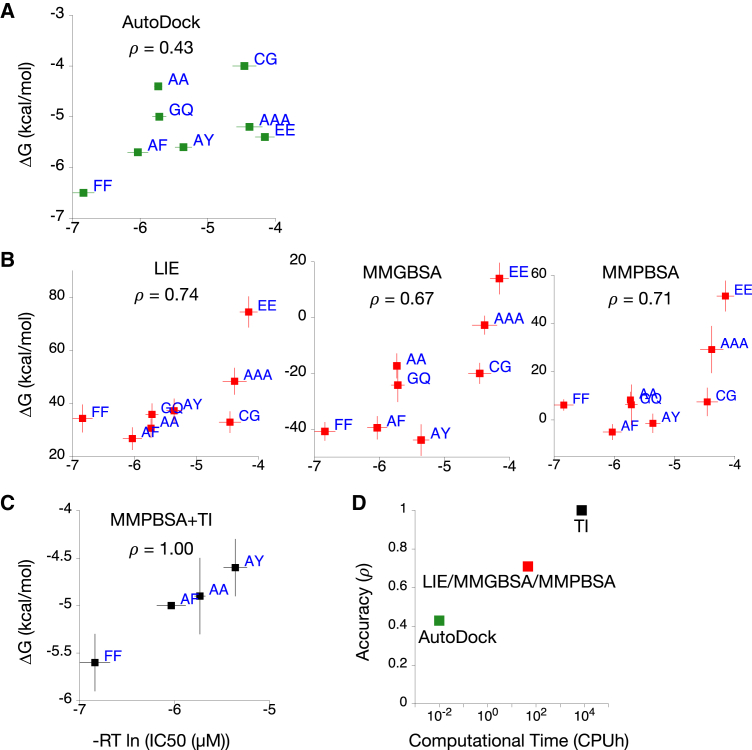
Endpoint and Exact Free Energy Methods can Predict Affinities Accurately (A–C) Predicted ΔG from free energy methods: (A) scoring functions (fast method), (B) endpoint methods (intermediate methods), and (C) theoretically exact method (slow method), compared with IC_50_ values from transport assay. The magnitudes of ΔG obtained from endpoint methods are significantly larger than expected due to the absence of an entropic term in the calculation. Spearman's correlation coefficient, ρ, was calculated to measure the ability of each method to reproduce the same ranking as experimental data. Y error bars indicate statistical errors from de-correlated and equilibrated ΔG data during MD simulations, while X error bars indicate the standard deviations from triplicate experiments. (D) The performance of all prediction methods and the computational cost based on a quad core processor. AutoDock does not require any simulation and each docking protocol takes around 30 s, and therefore is plotted as 10^−2^ CPUh. Standard one-letter code abbreviations have been used for all di- and tri-peptides.

To determine which computational methods can best predict the binding of di- and tripeptides to PepT_St_, we needed a test set of peptides and a selection of computational methods to validate. For the test set, we chose seven dipeptides and one tripeptide (triAla) for which experimental transport data were available. Crystal structures of one dipeptide (AlaPhe) and one tripeptide (triAla) bound to PepT_St_ are known (PDB: 4D2C, 4D2D, respectively; Lyons et al., 2014). The pose of the other six dipeptides was assumed to be the same as AlaPhe, as illustrated in Figure 1. We then selected a range of computational methods for calculating binding free energies which we would validate using the test set. The methods can be categorized based on the amount of computational resource each requires (Figure 2D); at the low end is the structure-based scoring function found in AutoDock Vina (Trott and Olson, 2010). Next we chose three different endpoint methods: the linear interaction energy (LIE; Aqvist et al., 1994), molecular mechanics generalized Born surface area (MMGBSA; Onufriev et al., 2000), and molecular mechanics Poisson Boltzmann surface area (MMPBSA; Kollman et al., 2000). All of these require some molecular dynamics (MD) simulation and hence are more expensive. Finally, we selected a theoretically exact method, thermodynamic integration (TI; Kirkwood, 1935) to calculate differences in binding free energies (ΔΔG) to refine the other predictions. Experimental binding data for PepT_St_, and POT transporters in general remain scarce, and the standard method for estimating affinities is to perform competition transport assays and measure the half maximal inhibitory concentration (IC_50_) values (Solcan et al., 2012). Unlike certain enzymes, however, transporters have more complicated kinetics such that the relationship between IC_50_ and ΔG is unclear (Eraly, 2008). We therefore compared these two datasets in a qualitative manner using Spearman's correlation coefficient (Lehmann and D'Abrera, 1998), ρ, which assesses the ability of each computational approach to reproduce the ranking of substrates based on experimental IC_50_ values.

Since it does not require any MD simulations, the scoring function is the fastest method to estimate binding affinities. Our results with AutoDock (Figure 2A), however, shows that it is also the least accurate (ρ = 0.43). This is not surprising as AutoDock uses a simplified scoring function (Wang et al., 2002). Although not tested in this study, it is possible that other scoring functions may produce better predictions for peptide transporters as no single docking program performs best across all protein families (Warren et al., 2006, Ross et al., 2013). Also, AutoDock does not account for the conformational sampling of the ligand and the residues in the binding site of the protein as it uses only one snapshot of the protein-ligand complex for its calculation. It is worth noting that this may be improved by using multiple conformers of the complex, for example from MD simulations, as has been done with several other membrane transporters (Schlessinger et al., 2011, Geier et al., 2013). As the binding of the peptide test set was modeled using the same structure, AutoDock Vina predicted that they all have very similar ΔG values. For each dipeptide, the range of ΔG values predicted for the nine poses generated is small (∼0.5 kcal/mol), although the score for the pose most similar to the crystal structure or homology model is not always the highest (Figure S1). We therefore conclude that AutoDock Vina does not accurately predict peptide-binding affinities for PepT_St_.

Encouragingly, all three endpoint free energy methods managed to rank the peptide test set well (Figure 2B) compared with the experimental data (ρ ≈ 0.7). The predicted ΔG values for the eight peptides span a wider range, allowing us to better distinguish the subset of well-transported peptides (PhePhe, AlaPhe, AlaAla, and AlaTyr) from poorly transported peptides (triAla and GluGlu). We assume that this increase in accuracy is primarily a result of using an ensemble of conformations generated during the MD simulation, which accounts for the conformational sampling of the ligand and the protein. As endpoint methods require only simulations of the bound and unbound states, the computational cost required for each calculation is relatively modest and therefore they are suitable candidates for a high-throughput workflow to differentiate between high-from low-affinity peptides.

Upon closer examination, however, we found that the endpoint methods poorly ranked peptides with similar IC_50_ values, for example the ρ value of the MMPBSA methods for hydrophobic dipeptides with IC_50_ ≤ 100 μM is 0.0, i.e., random (Figure S2). We hypothesized that the more rigorous method, TI might improve the ranking of AlaPhe, AlaAla, AlaTyr, and PhePhe by calculating the change in ΔG (ΔΔG) when the amino acids in AlaAla were mutated into either Phe or Tyr. These values were subsequently added to the results of the endpoint methods. We found that by implementing this step, we managed to significantly improve the prediction and reproduce the exact experimental ranking (ρ = 1.0) (Figure 2C). It is acknowledged, however, that due to the few data points, the apparently higher correlation to experimental data may be artificial.

To quantify and compare the exact amount of resources required for each prediction method, computational usage in hours of single CPU usage (CPUh) was estimated based on the performance of GROMACS for MD simulation using an Intel quad core Xeon processor (Figure 2D). It is no surprise that the more computational input fed into the methods, the more accurate the predictions become. The endpoint methods are an excellent compromise between good performance and low cost. We therefore conclude that it is most efficient to adopt a hybrid approach: using endpoint methods to broadly classify the ligands into high- and low-affinity substrates and then applying TI where necessary to further refine specific predictions.

### Binding of Dipeptides Sensitive to their N-Terminal Residue

**Figure 3:**
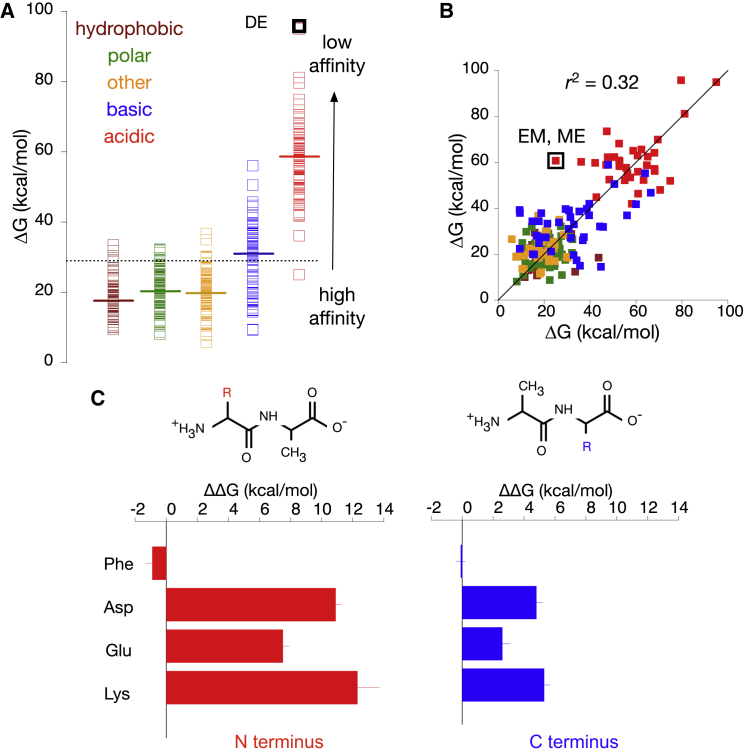
Further Predictions Suggest that the N-Terminal Side Chain Contributes More Toward the Binding of a Dipeptide than the C Terminus (A) ΔG values for all 400 dipeptides as predicted by the LIE method, arranged according to the overall chemical property of the peptide. Solid lines indicate the average of ΔG for each category and the dotted line represents the overall mean ΔG value. The magnitudes of ΔG for all peptides were predicted to be above zero due to the lack of entropic term in the calculation. (B) ΔG values plotted in pair, each for two dipeptides made of the same residues but in different orders, e.g., GluMet and MetGlu. The line drawn on the graph represents perfect linear correlation. (C) The TI method was used to calculate ΔΔG of alchemically changing the side chain of either the N or C terminus of AlaAla into Phe, Asp, Glu, or Lys. Errors were calculated by dividing all simulations into an equal number of independent bins as indicated by the reverse cumulative averaging method (Yang et al., 2004).

Having determined, using the test set of peptides, which free energy methods are most accurate, we now make some predictions for the bacterial transporter PepT_St_ and test them experimentally. Since the LIE method ranked the test set the best, we used it to predict ΔG for all possible 400 dipeptides. We again assumed that all dipeptides bind in the same orientation as AlaPhe, as has been elucidated by Lyons et al. (2014). In agreement with previous studies on PepT1 (Vig et al., 2006), PepT2 (Biegel et al., 2006), and a peptide transporter from *Saccharomyces cerevisiae*, Ptr2p (Ito et al., 2013), our results predict that neutral dipeptides made of hydrophobic and polar amino acids are the preferred substrates for PepT_St_ with almost all binding free energies below average (Figure 3A). As expected, we also found that acidic dipeptides bind least well with the lowest affinities predicted if both side chains are negatively charged, e.g., AspGlu. However, our results suggest that dipeptides with basic residues have moderate affinities for PepT_St_, while an experimental transport assay showed that LysLys has a high IC_50_ value, indicating poor transport (Solcan et al., 2012).

Previous modeling of PepT1 suggested that the binding site predominantly recognizes the peptide backbone and therefore substrate affinities should not be significantly affected by the sequence of residues (Foley et al., 2010). To check if our results agreed with this prediction, we replotted each ΔG value against its sequence-reversed equivalent, i.e., Ala-X versus X-Ala (Figures 3B and S3). If order does not matter, then the points should all fall on a straight line; however, we found a relatively low correlation (r^2^ = 0.32) with some pairs differing significantly, for example, AlaLys and LysAla. Our results therefore suggest that the order of amino acid residues does influence the overall affinity in PepT_St_.

To explore this further, TI calculations were performed to determine how the binding free energy changes when the N- or C-terminal side chain of AlaAla is transmuted into either phenylalanine, aspartate, glutamate, or lysine (Figure 3C). Negative ΔΔG values (and therefore better binding) were obtained when either side chain was substituted with Phe, exemplifying the inclination of this transporter toward hydrophobic peptides as shown by other POTs (Gebauer and Hartrodt, 2003, Biegel et al., 2006, Doki et al., 2013, Fang et al., 2000). Transformation into a charged residue, however, resulted in a reduction of binding affinity (positive ΔΔG), in agreement with our LIE predictions. In all cases, when the N terminus was altered, the magnitude of the change in binding affinity was larger than the identical change at the C terminus. These results indicate that the N-terminal residue is crucial in determining the selectivity of PepT_St_.

We then investigated which residues in the binding site of the transporter interact with the side chains of the dipeptides (Figure S4). The C-terminal side chain mainly occupies a hydrophobic pocket and forms hydrophobic interactions primarily with Tyr68 and Trp296. The N-terminal side chain, however, contacts a group of polar residues including Tyr30, Asn156, and Asn328. All of these residues have been previously shown to be important for peptide transport by PepT_St_ (Solcan et al., 2012). These analyses suggest that the N-terminal side chain potentially binds more strongly to the binding site via multiple electrostatic interactions and hydrogen bonds, and thereby is more sensitive to changes, whereas the C-terminal counterpart is accommodated inside a hydrophobic pocket that can adapt to various chemical groups without a large energetic penalty, and therefore is less sensitive when perturbed to another side chain.

**Figure 4:**
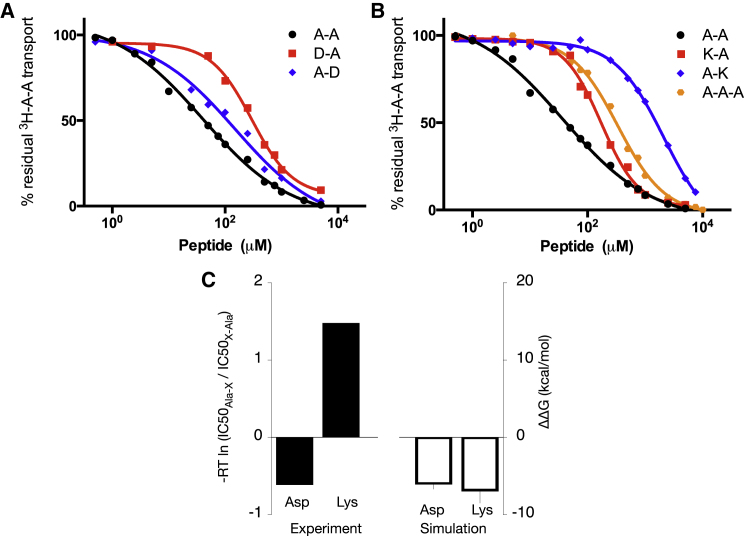
Proton-Driven Competition Uptake Assays (A) IC_50_ competition curves for AlaAsp and AspAla showing residual uptake of ^3^H-AlaAla in a proteoliposome-based transport assay. (B) Similar IC_50_ competition curves for AlaLys, LysAla, and triAla. (C) Apparent “ΔΔG” values derived based on the ratio of IC_50_ of Ala-X and X-Ala, compared with the corresponding calculated ΔΔG values from simulation. Errors were calculated as per Figure 3.

To test these predictions, we performed proton-driven competition uptake assays for AlaAsp, AspAla, AlaLys, and LysAla using radiolabeled AlaAla as the reporter substrate. The results were then compared with the uptake of the neutral peptides, AlaAla and triAla (Figures 4A and 4B). To make comparison with our in silico predictions easier, we determined the ratio of IC_50_ values between AspAla and AlaAsp as well as between LysAla and AlaLys, and calculated the apparent “ΔΔG” values (Figure 4C). Consistent with our in silico predictions, having the aspartate residue at the N terminus is more detrimental to transport compared with having the same residue at the C terminus, as exemplified by the larger IC_50_ of AspAla (300 μM) compared with AlaAsp (100 μM) and therefore the negative apparent “ΔΔG” value. A similar experiment for positively charged dipeptides, AlaLys and LysAla, however, showed the opposite trend. LysAla has a lower IC_50_ than AlaLys, i.e., 150 μM and 2.1 mM, respectively, and their apparent “ΔΔG” value is therefore positive. This indicates that for lysine, positioning this residue on the C terminus of a dipeptide is more detrimental to its affinity.

### Lysine-Containing Dipeptides are Predicted to Bind in a Tripeptide-like Pose

**Figure 5:**
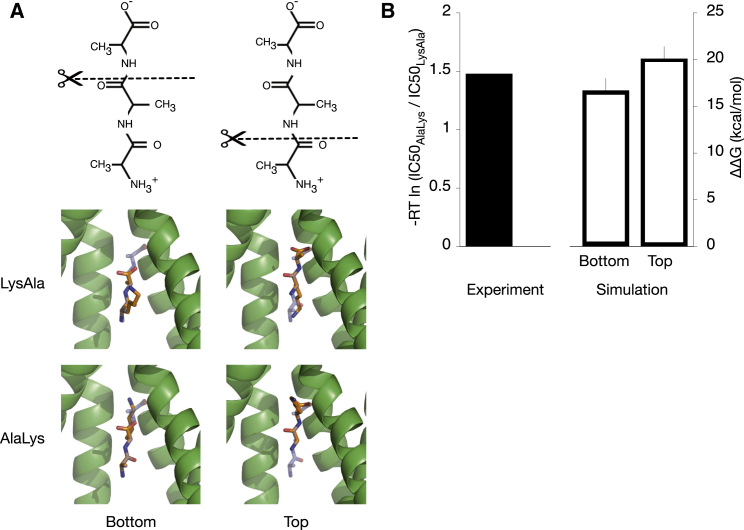
Re-Modelling the Binding of LysAla and AlaLys Based on a Tripeptide, triAla (A) Two ways to model the binding of a dipeptide based on triAla: (1) by removing the C-terminal residue, and using the first and second residues as template (bottom models) and (2) by removing the N-terminal residue, and using the second and third residues as template (top). The figures overlay the new models of AlaLys and LysAla (orange stick representation) on top of triAla (purple). (B) The TI method was used to calculate ΔΔG of alchemically transforming the N- or C-terminal side chain of AlaAla (in both bottom and top models) into lysine. These are compared with the apparent “ΔΔG” values derived based on the ratio of IC_50_ of AlaLys and LysAla from the transport assays. Errors were calculated as per Figure 3.

The discrepancy between our computational predictions and experimental data for positively charged dipeptides could be as a result of many different factors such as inadequate sampling and force field errors. One other possible reason is that these peptides do not bind in the same horizontal pose as AlaPhe. As the vertical pose is the only alternative binding mode observed in crystal structures (Figure 1B), we explored this hypothesis by modeling the binding of AlaAla, AlaLys, and LysAla based on the crystal structure of PepT_St_ bound to triAla (PDB: 4D2D; Lyons et al., 2014). TriAla has three residues, so there are two possible ways of modeling a dipeptide: (1) removing the C-terminal residue and using the first and second residues as the template or (2) removing the N-terminal residue and using the second and third residues as the template. The former approach positions the model dipeptide closer to the cytoplasmic side, and henceforth is called the bottom model, whereas the latter places the peptide nearer to the extracellular side (top model) (Figure 5A). As before, we performed TI to alchemically convert either the N- or C-terminal side chain of AlaAla in this vertical binding modes into lysine, and subsequently calculated how the difference in binding free energy (ΔΔG) between LysAla and AlaLys.

Our results for both the bottom and top models suggest that AlaLys has a less negative ΔG value and thereby binds less tightly compared with LysAla, as suggested by the positive ΔΔG (Figure 5B), in agreement with previously performed transport assays. Further inspection of the alternative binding models suggests that the strong binding of LysAla stems from favorable salt bridges formed via the ε-amino group with Tyr68 and Glu300 in the bottom model (Figure S5A) or Asn156 in the top model (Figure S5B). In contrast, the lysine side chain of AlaLys protrudes into a cavity that is in close proximity to Arg33, therefore resulting in unfavorable electrostatic repulsion and hence a more positive ΔG value (Figure S5D).

While our modeling appears to suggest that two possible vertical binding sites exist for a dipeptide, i.e., the bottom and top models, we acknowledge that in reality this might not be the case. The binding of triAla to PepT_St_ in the original crystal structure showed fewer contacts with residues in the binding cavity compared with AlaPhe, indicating a weak interaction, consistent with the higher IC_50_ value (Lyons et al., 2014). Similarly, in our simulation, a ligand bound in this orientation was able to move up and down, suggesting that there are no distinct “bottom” and “top” binding sites, instead there is just one large vertical cavity. Hence, one would expect to obtain similar values of ΔΔG for both models. That this is not the case suggests the TI simulations have not converged, which is not surprising given the likely timescale for the ligand to explore the vertical pocket. Despite this, as they both show the same trend as experiment, our current results suggest that AlaLys and LysAla bind in a similar orientation to triAla, rather than the horizontal pose of AlaPhe. Nevertheless, further high-resolution crystal structures are still required to verify the validity of our prediction for these peptides.

### Experimental Structures are Essential for Accurate Predictions

A primary goal of this study is to develop a transferable approach of relevance to drug discovery. We therefore tried our method on a homology model of the pharmaceutically relevant human PepT1 (Beale et al., 2015) and expanded the test set to include 14 drug compounds that are known substrates of this transporter. Peptides were assumed to bind to PepT1 in the same way as to PepT_St_. The binding of drugs was modeled based on the conformations of either AlaPhe or triAla according to their size. Our predicted ΔG values were then compared with the IC_50_ data from whole-cell transport assays (Biegel et al., 2005, Vig et al., 2006). Unfortunately, all methods failed to distinguish between good and poor binders with correlation coefficients of ∼0.0 (indicating that the predictions are random) (Figure S6).

**Figure 6:**
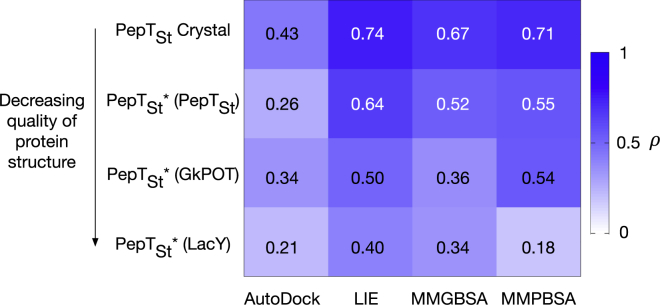
Comparison of Performance of Binding Affinity Predictions Using a Scoring Function (AutoDock) and Endpoint Methods, LIE, MMGBSA, and MMPBSA, for Homology Models of PepT_St_ as Marked with Asterisks The template used for each model is shown in parentheses. The number in each box represents Spearman's correlation coefficient, ρ, between predicted ΔG and experimental IC_50_ data for a test set of eight peptides (as shown in Figure 2). These are colored from white (0.0) to blue (1.0).

One potential reason for this loss of predictive ability is the lack of a high-resolution crystal structure of human PepT1. To investigate how much the approach depends on the quality of the protein structure, a series of homology models for the bacterial homolog PepT_St_ were built using as templates, in decreasing order of quality: (1) the crystal structure of PepT_St_ itself (PDB: 4D2C and 4D2D), (2) the crystal structure of GkPOT (PDB: 4IKV) that shares ∼50% sequence identity with PepT_St_, and (3) the crystal structure of LacY (PDB: 1PV6), which has only ∼25% sequence homology compared with PepT_St_. For the same peptide test set as before, all three homology models showed lower correlations for all methods compared with predictions using the crystal structure of PepT_St_. Interestingly, the predictive ability of all the endpoint methods degrades proportionally to the quality of the protein structure (Figure 6), with the best model showing a slight decrease in the ρ value, followed by larger decreases for the intermediate and poor-quality models. Taken together, these results suggest that, while our current approach can be applied to other members of the peptide transporter family, a high-resolution crystal or electron microscopy structure is essential to achieve accurate results.

## Discussion

We have demonstrated that computational modeling, molecular simulation, and free energy calculations can accurately predict protein-ligand interactions for a membrane transporter. Using an endpoint method, LIE, we first classified all 400 possible dipeptide ligands into strong and weak binders. In agreement with experimental data from other peptide transporters (Vig et al., 2006, Biegel et al., 2006, Ito et al., 2013), we found that uncharged peptides are, on average, the best substrates, while acidic residues bind weakly. Refining these results using a theoretically rigorous method, TI, we then found that the N-terminal residue of a dipeptide contributes significantly toward selectivity. These predictions were tested experimentally, which showed good agreement except for basic dipeptides. Remodeling the basic peptides based on the vertical binding orientation restored agreement between simulation and experiment, therefore we hypothesize that these basic dipeptides may not interact with PepT_St_ in the canonical dipeptide-binding pose, but instead possibly mimic a tripeptide.

It is important at this stage to highlight the limitations of our computational approach. The MD simulations were performed using a molecular mechanics force field, which is a classical approximation of a more complex quantum mechanical reality at the atomic level. It is well established, however, that this force field is accurate at reproducing many experimental results (Shirts et al., 2003). The magnitude of the absolute ΔG values calculated by the endpoint methods are large and mostly positive, rather than small and negative as one would expect (Figures 2B and 3A). This is due to various assumptions we have made. The MMGBSA and MMPBSA methods neglect the entropic contributions, ΔS, from the protein and ligand, although we expect the change in entropy to be similar since all of the ligands are structurally similar (Oehme et al., 2012). In principle, one could estimate the change in entropy; however, the currently available methods are not precise and return very large standard errors (Kar et al., 2011). For the LIE method, we implemented hydroxyl-based scaling factors (Hansson et al., 1998) for simplicity, instead of the more sophisticated parametrizations that take into account other chemical groups (Wang et al., 1999, Almlof et al., 2007). While these computed ΔG values should not be considered as the true binding free energy, they give meaningful results when used qualitatively, for example, in the comparison between various ligands to understand the general trend of binding affinity. Our modeling is also limited to the inward open conformation, so the ΔG calculated might not be representative for other conformations of the transporter. Despite these limitations, our predictions for PepT_St_ using the peptide test set show good agreement with experimental data, suggesting that these methods are indeed sufficient to study ligand interactions.

A ligand-based substrate template of PepT1 (Bailey et al., 2000) suggested that dipeptides are better substrates than tripeptides. However, this template fails to account for the generally poor transport behavior of basic dipeptides that show worse affinities than neutral tripeptides (Eddy et al., 1995, Terada et al., 2000). Here, we propose based on computer modeling and competition assays that the lower affinities of these dipeptides arise from a different binding pose. Unlike neutral and acidic dipeptides, a dipeptide with lysine residues is proposed to bind like a tripeptide in a vertical binding orientation, which results in poorer transport as this binding mode is less tightly coordinated (Lyons et al., 2014). The large extended lysine side chain makes the total length of AlaLys and LysAla ∼10 Å, which is about the same as the backbone of a tripeptide. As such, positioning these dipeptides laterally like AlaPhe may cause unfavorable steric clashes and the only way for them to bind is by treating their large side chain effectively as an additional residue. As the side chain of an arginine residue is of similar length and is also capped by a positive charge, we conjecture that dipeptides with arginine may also interact this way, which would explain their poor uptake.

**Figure 7:**
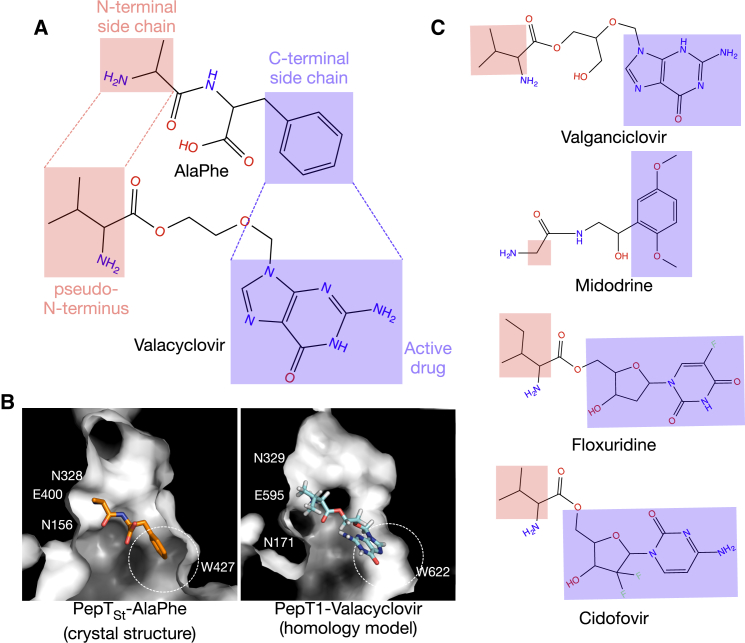
A Model of Peptide Prodrug Binding to PepT1 (A) Structural comparison of an antiviral prodrug valacyclovir and AlaPhe suggests that the attached amino acid, valine (red), acts as a pseudo N terminus, whereas the active drug, acyclovir (blue), mimics the position of the second residue of a dipeptide. (B) Surface representation of the binding site of PepT_St_-AlaPhe (crystal structure) and PepT1-valacyclovir (homology model) highlighting the position of the phenylalanine side chain and acyclovir inside a hydrophobic cavity, depicted by dotted circles. (C) Other prodrugs that target PepT1 following the approach of adding an amino acid residue to form an N terminus.

The importance of the N terminus for peptide binding to PepT1 has been demonstrated by previous studies (Borner et al., 1998, Meredith et al., 2000), which suggested that the amino group is responsible for aligning the rest of the molecule in the central binding cavity. Similar results were observed for Ptr2p (Ito et al., 2013), where the N-terminal residue showed a higher propensity toward determining whether a substrate belongs to a high- or low-affinity group. Our studies lend further support to this idea by showing that the side chain on the N terminus is the primary determinant of binding selectivity in PepT_St_. The structural reasoning behind this observation is that this side chain contacts a group of polar residues in the binding site via multiple electrostatic interactions, while the C-terminal side chain is surrounded by an electro-neutral binding cavity. As PepT_St_ and human PepT1 share 80% sequence identity within the peptide-binding site, it is likely that PepT1 has a similar substrate recognition mechanism. This would explain why the peptide prodrug approach targeting PepT1 has been very successful. These prodrugs are structurally analogous to a dipeptide, and the added amino acid acts as a pseudo N terminus and the active drug moiety the C terminus (Figure 7). While the N-terminal amino acid is crucial to gain affinity to the transporter, the drug compound itself fits well inside the large hydrophobic cavity, and hence the prodrugs are recognized and transported by PepT1. This prodrug recipe has been employed for various drugs such as the antivirals valacyclovir (Ganapathy et al., 1998), valganciclovir (Sugawara et al., 2000), and cidofovir (McKenna et al., 2005).

Generating a substrate-binding model for a membrane transporter presents enormous possibilities for rational drug design. Previous studies of the mammalian PepT1 transporter produced a simplified two-dimensional model (Meredith et al., 2000), followed later by three-dimensional pharmacophores (Biegel et al., 2005, Vig et al., 2006, Foley et al., 2010, Pedretti et al., 2008). A more detailed structure-based model is imperative following the recent crystallographic and thermodynamic evidence that this transporter operates with at least two distinct binding modes (Parker et al., 2014). We made the best use of these data by combining various computational techniques alongside experimental transport assays to suggest how other peptides and drugs interact with this transporter. While it is currently not possible to directly apply our methods to the human PepT1 due to the lack of a high-resolution crystal structure, our promising results with its model system, PepT_St_, demonstrate its potential for the study of drug-transporter interactions. With the ever-increasing computational power and available crystal structures of membrane transporters, we expect this approach to be extended to other relevant transport systems and larger compound libraries in the near future.

## Significance

**When a drug is taken orally, it has to pass through the lining of the gut to enter the blood circulation and reach its target. Membrane proteins found in the gut epithelium, for example, the human peptide transporter, PepT1, play key roles in mediating the absorption of orally prescribed drugs. Designing drugs that can strongly bind to PepT1 will therefore improve bioavailability, and in turn optimize dosing and minimize undesirable side effects. Here, we demonstrate an accurate way to computationally determine how well a ligand binds to a bacterial homolog of PepT1. This approach is capable of discriminating strong and weak binders and elucidating the origin of ligand selectivity in this transporter, which we then validate using in vitro transport assays. Studying drug-transporter interactions is becoming an important component of rational drug design. We foresee that this method will be more widely employed in the future to study other medically relevant membrane transporter families.**

## Experimental Procedures

The binding of both peptides and drugs was modeled based on the structure of PepT_St_-AlaPhe (PDB: 4D2C) and PepT_St_-triAla (PDB: 4D2D) complexes (Lyons et al., 2014). For predicting the binding free energy, ΔG, using a scoring function (AutoDock Vina; Trott and Olson, 2010), each peptide and drug compound was docked to the binding site of the protein and the affinity score from the pose most similar to the crystal structure (for AlaPhe and triAla) or model (for other ligands) was taken as the ΔG value. For ΔG predictions using the endpoint methods, 1-ns MD simulations were performed using GROMACS 4.5.4 (Hess et al., 2008) with parameters explained in detail in the Supplemental Information. We performed two simulations for the LIE method: (1) a protein-ligand complex in membrane and (2) the ligand in bulk solution. The GROMACS tool, g_lie, was used to compute ΔG values with the α scaling constant set to 0.18 (Luzhkov and Aqvist, 2001, Osterberg and Aqvist, 2005) and the β scaling constant set to either 0.5, 0.43, 0.37, or 0.33 based on the number of hydroxyl groups on the ligand molecule (Hansson et al., 1998). For MMGBSA and MMPBSA, the single trajectory protocol was used (Hou et al., 2011), where only the simulation of protein-ligand complex was run and the trajectories of unbound protein and free ligand were extracted from it. The calculation of ΔG was done using the MMPBSA.py program (Miller et al., 2012) with the implicit solvation parameters (saltcon and istrng) set to 0.15 M.

For predictions of ΔΔG by the TI method, alchemical MD simulations were performed using either the PepT_St_-AlaPhe crystal complex or PepT_St_-AlaAla model for the starting coordinates. The dual topology approach was employed where both the vanishing and growing atoms were represented separately. All transformations involved only the non-bonded interactions, while bonded interactions were kept the same throughout the simulations (Boresch and Karplus, 1999, Boresch, 2002). Two set of MD simulations were run: (1) the transformation of peptide ligand bound to PepT_St_ (bound state) and (2) the transformation of peptide ligand in bulk solution (unbound state). The transformation of both states was divided into three steps: (1) removing the partial charges of the disappearing chemical groups, (2) removing the van de Waals interactions of the disappearing groups while adding that of the emerging groups, and (3) adding the partial charges of the emerging groups. A soft core potential (Beutler et al., 1994) was applied in step (2) to avoid singularities and instabilities. These transformations were coupled to a scaling parameter, λ, where at λ = 0, the non-bonded terms of the initial peptide were used, while at λ = 1, the non-bonded terms of the final peptide were used. Eleven independent 5-ns MD simulations were performed at λ = 0, 0.1, 0.2, 0.3, 0.4, 0.5, 0.6, 0.7, 0.8, 0.9, 1. The trapezoid rule was used to integrate the ∂U/∂λ values to obtain ΔG. In the transformations with a change in the charge of the ligand, the one-box approach described by Rashid et al. (2013) was employed.

The performance of each prediction method was assessed by Spearman's rank correlation coefficient (Lehmann and D'Abrera, 1998) using the following equation:

$$\text{ρ}=1-\frac{6\sum {\left(x_{i}-y_{i}\right)}^{2}}{n\left(n^{2}-1\right)},$$

where x_i_ is the ranking for experimental IC_50_ values, y_i_ is the ranking for predicted ΔG values and n is the size of the dataset. To validate our computational predictions, proteoliposome-based competition transport assays were performed as described in Solcan et al. (2012).

## Author Contributions

F.S., M.S.P.S., and P.W.F. conceived the project, designed experiments, and wrote the manuscript. F.S. performed computational predictions of ligand affinities with assistance from P.W.F. J.L.P. and S.N. performed experimental transport assays. All authors reviewed the paper.

## Supplemental Information

Document S1. Supplemental Experimental Procedures and Figures S1–S6

Document S2. Article plus Supplemental Information
