# Supplementary material for: Accurate Prediction of Ligand Affinities for a Proton-Dependent Oligopeptide Transporter
Source: Cell Chem Biol. 2016 Feb 18;23(2):299–309. doi: 10.1016/j.chembiol.2015.11.015 (PMC4760754; doi:10.1016/j.chembiol.2015.11.015)
Supplement: Document S1. Supplemental Experimental Procedures and Figures S1–S6 [file mmc1.pdf]

Cell Chemical Biology, Volume *23*

Supplemental Information

Accurate Prediction of Ligand Affinities for a Proton-Dependent Oligopeptide Transporter

Firdaus Samsudin, Joanne L. Parker, Mark S.P. Sansom, Simon Newstead, and Philip W. Fowler

Accurate prediction of ligand affinities for a peptide transporter – Supplemental Information

Firdaus Samsudin, Joanne L. Parker, Mark S. P. Sansom, Simon Newstead,* and Philip W. Fowler†

Department of Biochemistry, University of Oxford, South Parks Road, OX1 3QU

Contents

1 Supplemental Figures **2**

2 Supplemental Experimental Procedures **9**

2.1 Modelling peptide and drug binding 9

2.2 Molecular dynamics simulation 9

2.3 Binding energy predictions 10

2.4 Competition transport assays 13

List of Figures

S1 The 9 poses generated by AutoDock Vina for the dipeptide AF. . . 3

S2 Binding affinity predictions refined by thermodynamic integration . 4

S3 Predicted ΔG values from the LIE method. 5

S4 The N-terminus side chain interacts with a polar cavity while C-terminus side chain occupies a hydrophobic pocket. 6

S5 Interactions of the lysine side chains of AlaLys and LysAla with residues in the binding site of $\mathrm{PepT_{St}}$. 7

S6 Binding affinity predictions for a homology model of PepT1. . . . 8

*E-mail: simon.newstead@bioch.ox.ac.uk; Corresponding author

†E-mail: philip.fowler@bioch.ox.ac.uk; Corresponding author

1 Supplemental Figures

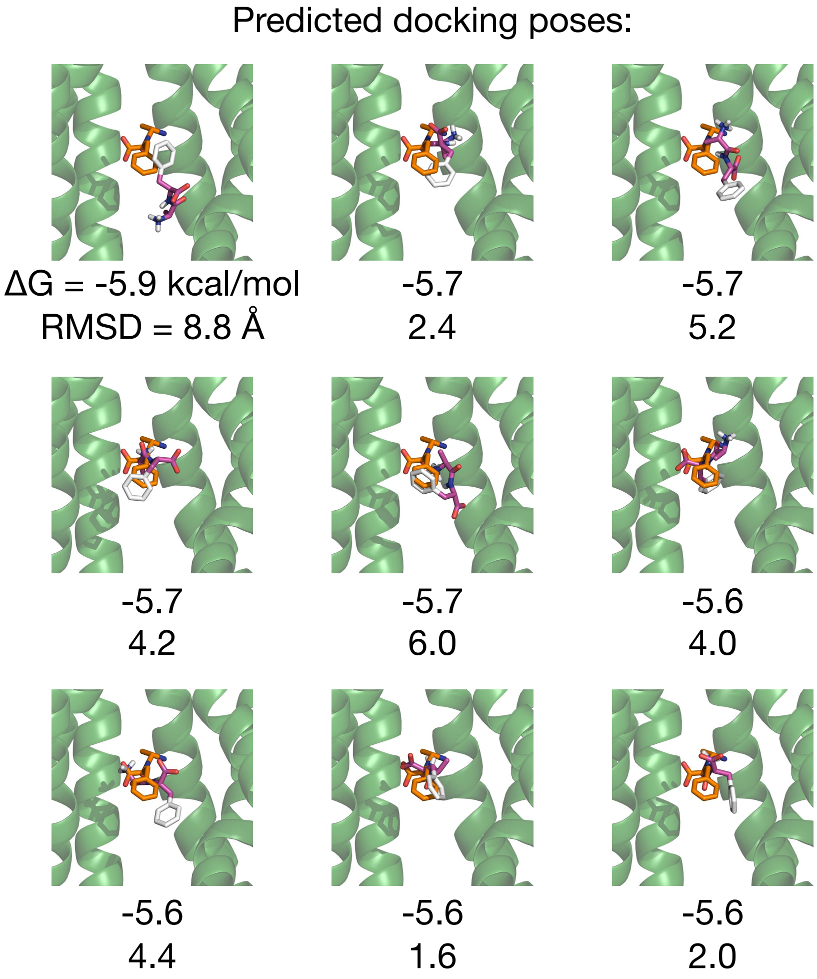

Figure S1: related to Figure 2 - The nine docking poses generated by AutoDock Vina for the dipeptide AlaPhe. These poses are arranged based on ΔG estimated by a scoring function. The predicted binding orientations (pink) are compared to the crystal structure (orange) and the all-atom RMSD is shown.

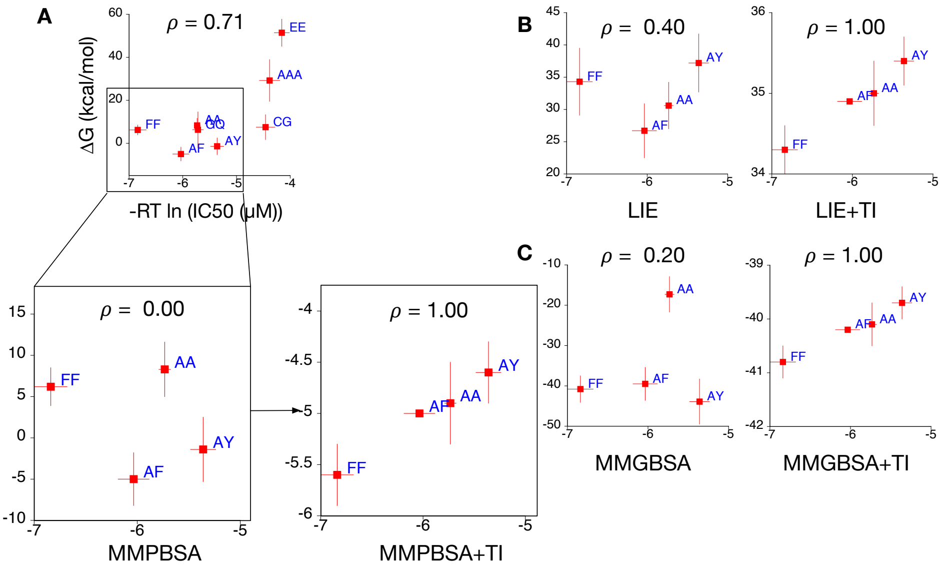

Figure S2: related to Figure 2 - Binding affinity predictions refined by thermodynamic integration. (A) End-point free energy methods like MMPBSA fails to discriminate amongst the good substrates (IC50≤100 μM) as shown in the enlarged image. TI was employed to calculate $\Delta\Delta$G of these peptides with respect to AlaPhe, and subsequently used to adjust the MMPBSA results. Y-error bars indicate statistical errors from de-correlated and equilibrated ΔG data during MD simulations, while X-error bars indicate the standard deviations from triplicate experiments. The line drawn on each graph represents the least square fit. (B) and (C) illustrate similar refinement steps for LIE and MMGBSA, respectively.

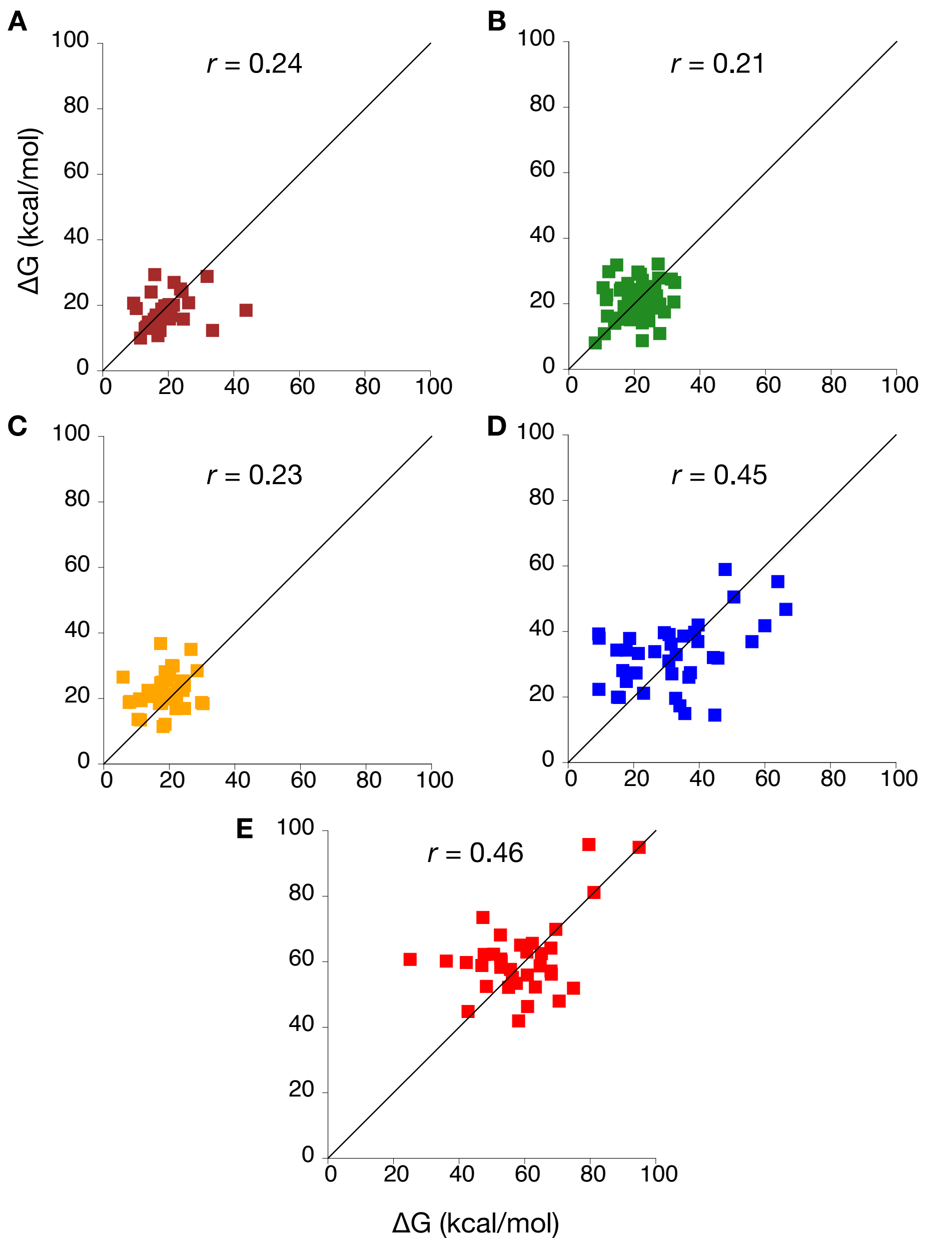

Figure S3: related to Figure 3 - Predicted ΔG values from the LIE method plotted in pairs based on residue combinations, whereby each pair of peptides are made of the same combination of amino acids but in different orders. They are plotted according to the overall chemical properties of the peptides: (A) hydrophobic, (B) polar, (C) other, (D) positive and (E) negative.

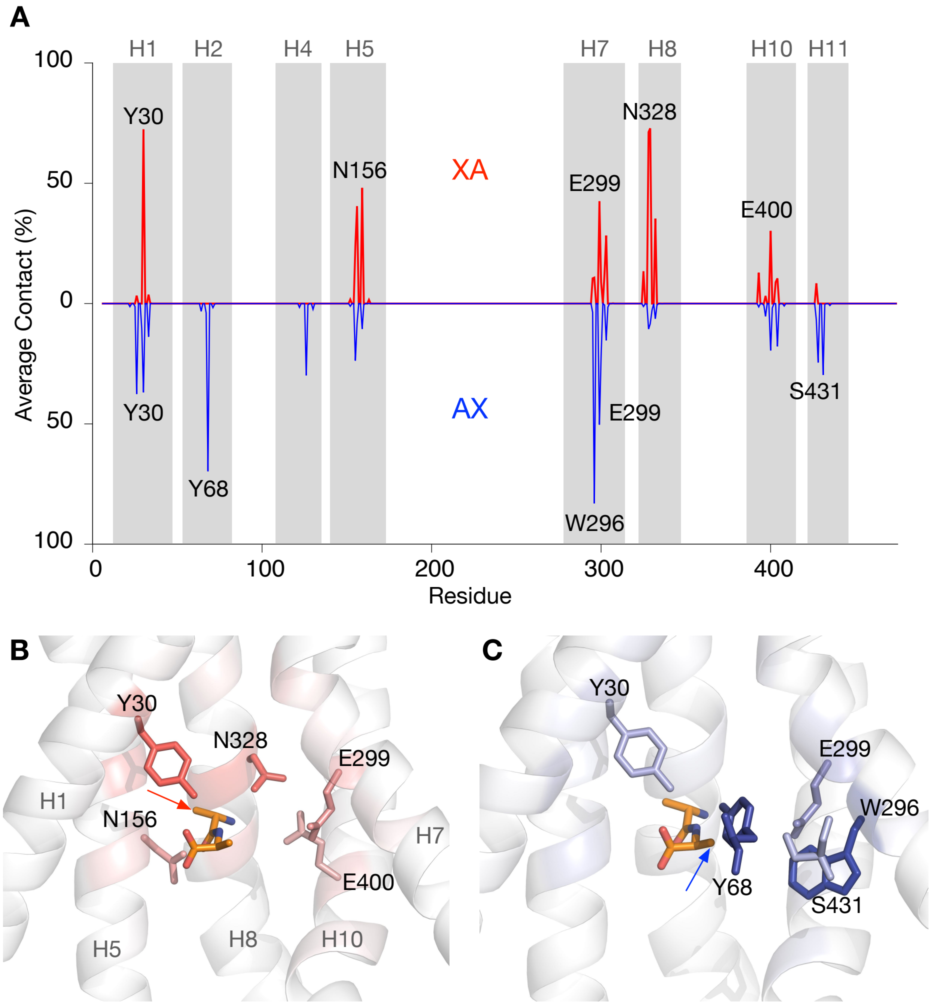

Figure S4: related to Figure 3 - The N-terminus side chain interacts with a polar cavity while C-terminus side chain occupies a hydrophobic pocket. (A) Analysis of contacts made by the side chain of dipeptides Ala-X and X-Ala with $PepT_{St}$ during simulation, whereby X is one of the 20 amino acids. This is averaged over 20 simulations of length 1 ns (one for each amino acid). Residues that make significant contacts are mapped to the binding site ((B) and (C)), while arrows indicate the side chain of interest. The cut-off for contacts is set to 3.5 Å.

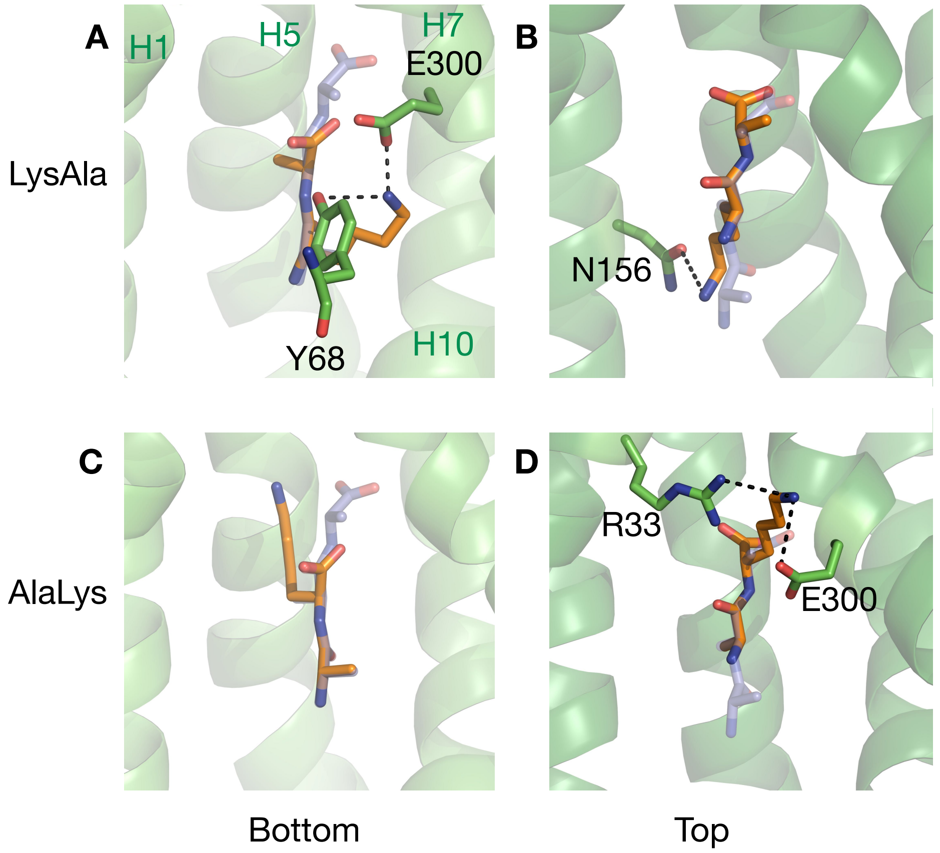

Figure S5: related to Figure 5 - Interactions of the lysine side chains of AlaLys and LysAla (orange stick representations) with residues in the binding site of $PepT_{St}$ (green). Figures show residues that are found within 4 Å of the ϵ-amino group of the side chain and can potentially interact via electrostatic attractions or repulsions. No residues were found in the vicinity of this group for the AlaLys Bottom model. The coordinates of triAla, on which these dipeptides were modelled, are represented in purple.

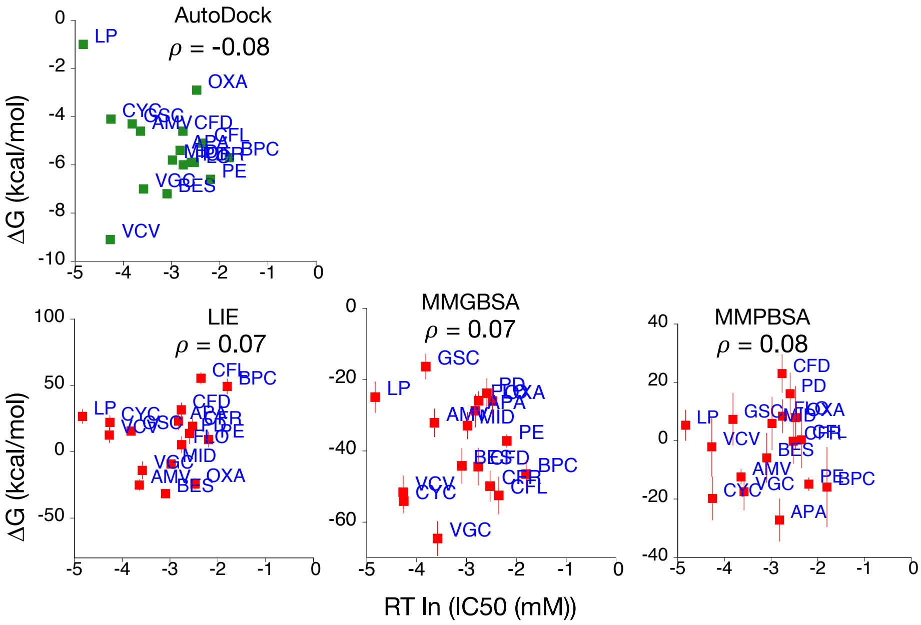

Figure S6: Figure S6, related to Figure 2 and Figure 6 - Extending binding affinity predictions to a homology model of PepT1. Predictions are made using a scoring function (green) and end-point methods (red) for the same peptide test set with additional 14 drug compounds (listed in Experimental procedure). The ability of each method to reproduce experimental ranking is represented as Spearman's correlation coefficient, ρ.

2 Supplemental Experimental Procedures

2.1 Modelling peptide and drug binding

Modelling of peptide substrates were performed using Modeller-9v9 (Sali and Blundell, 1994). The template used for all dipeptides was the crystal complex $PepT_{St}$-AlaPhe (PDB:4D2C) (Lyons et al., 2014). Additionally, the binding of AlaLys and LysAla to $PepT_{St}$ were also modelled based on the structure of $PepT_{St}$-triAla (PDB:4D2D) (Lyons et al., 2014). Only the side chains of the peptide substrate were altered, whereas the structure of the rest of the peptide and protein was kept identical to the template. Models with the highest discreet optimised protein energy (DOPE) scores (Shen et al., 2006) were chosen for MD simulations and ΔG predictions.

Fourteen additional drug compounds were used in the test set of PepT1 homology model: valacyclovir (VCV), cyclacillin (CYC), glycosarcosine (GSC), aminolevulinic acid (AMV), bestatin (BES), valganciclovir (VGC), midodrine (MID), cefadroxil (CFD), floxuridine (FLO), cefaclor (CFR), oxacillin (OXA), aminophenylacetic acid (APA), cefalexin (CFL), and benzylpenicillin (BPC). To model the binding of a drug compound, the structure of the drug was first determined by inspection if it resembles AlaPhe or triAla. Small drugs (AMV, APA, GSC) and prodrugs (VCV, VGC, MID, FLO) are structurally analogous to AlaPhe and were therefore modelled according to the structure of $PepT_{St}$-AlaPhe complex (PDB:4D2C). The β-lactam antibiotics (CFD, CFL, CFR, CYC, OXA, BPC) and BES are more similar to triAla and hence were modelled based on the structure of $PepT_{St}$-triAla complex (PDB:4D2D).

2.2 Molecular dynamics simulation

Transporter proteins and peptide ligands were parametrised using the standard Amber99SB force field (Lindorff-Larsen et al., 2010) while the drug molecules were parametrised using the general Amber force field (GAFF) (Wang et al., 2004). Peptide-bound transporter was embedded in a pre-equilibrated 381-molecule 1-palmitoyl-2-oleoyl-sn-glycero-3-phosphocholine (POPC) model membrane (Dickson et al., 2012) inside a simulation box of dimensions 10 nm $\times$ 13 nm $\times$ 15 nm using the `g_membed` protocol (Wolf et al., 2010). The simulation

box was hydrated by TIP3P water (Jorgensen et al., 1983) and neutralised by 0.15 M Na^+ and Cl^- ions. The steepest-descent protocol was used to minimise the system before being subject to a 1 ns equilibration simulation, during which heavy atoms of the protein and peptide substrates were positionally-restrained. This restraint was removed afterwards for a production MD simulation. Peptide-only simulations underwent the same protocol, albeit without the membrane and in a smaller simulation box (size 3 nm $\times$ 3 nm $\times$ 3 nm). The length of production MD simulations performed for end-point methods is 1 ns and for TI, 5 ns for each λ.

The temperature within the simulation box was kept at 310 K using the velocity-rescaling thermostat with a time constant of 0.1 ps (Bussi et al., 2007). The pressure was kept at 1 atm by semi-isotropic coupling to a Parrinello-Rahman barostat with a time constant of 1.0 ps and compressibility of 4.5×10^{-5} bar^{-1} (Parrinello, 1981). The lengths of all bonds in the system were constrained using the LINCS algorithm (Hess et al., 1997) and an integration time step of 2 fs was employed by the leap-frog algorithm to solve the Newton's equations of motion. Long-range electrostatic forces were calculated by the particle mesh Ewald (PME) method (Essmann et al., 1995) using a real space cut-off of 12 Å, whereas van der Walls interactions were cutoff at 12 Å. The energy data were written out every 1 ps. All simulations were performed using GROMACS 4.5.4 (Hess et al., 2008) and visualised in PyMOL (Schrödinger) and VMD (Humphrey and Dalke, 1996).

2.3 Binding energy predictions

For ΔG predictions using a scoring function, AutoDock Vina (Trott and Olson, 2010) was used to dock each peptide to the binding site. This procedure generates nine binding poses, each with an affinity score. The binding poses were compared to the crystal structures (for AlaPhe and triAla) and models (other peptides and drugs) and the most similar pose according to the RMSD was selected and its binding energy score is taken as the ΔG value. AutoDock Vina was chosen as it implements a scoring function based on X-Score, which has been shown to be the best at ranking ligands (Cheng et al., 2009). No MD simulations were required in this procedure.

To calculate ΔG using the end-point free energy methods, 1 ns MD simulations were performed for each peptide and drug compound. For LIE (Aqvist et al., 1994), two types of simulation were conducted: (i) PepT_{St} bound to a peptide and (ii) the peptide alone in solution. A GROMACS tool, `g_lie`, was used to compute ΔG values along the simulation trajectory based on the energy outputs of both simulations. A default value of 0.18 was used as the non-polar scaling constant, α (Luzhkov and Aqvist, 2001; Osterberg and Aqvist, 2005). For the polar scaling constant, β, we used the hydroxyl-based parameters as suggested by Hansson et al. (1998), where for charged peptides, β = 0.5, for neutral peptides with no hydroxyl group, β = 0.43, for neutral peptides with one hydroxyl group, β = 0.37 and for neutral peptides with two hydroxyl groups, β = 0.33. For MMGBSA and MMPBSA, we follow the single trajectory protocol (Hou et al., 2011), where only the simulation of PepT_{St} bound to a peptide was run and the trajectories of unbound PepT_{St} and free peptide were extracted from it. The `MMPBSA.py` Miller III et al. (2012) program in the AmberTools package, was used to calculate ΔG for both MMGBSA and MMPBSA methods. The salt concentration (`saltcon`) for the generalised Born (GB) calculations and ionic strength (`istrng`) for the Poisson Boltzmann (PB) calculations were both set to 0.15 M.

Alchemical transformation MD simulations were performed to calculate relative free energy of binding $\Delta\Delta$G by the TI method. For our validation step (Figure 2), the crystal structure of PepT_{St}-AlaPhe was used for the starting coordinates and the ligand is gradually morphed to either AlaAla, AlaTyr or PhePhe. The dual topology approach was employed, whereby the vanishing and growing atoms were represented separately. For example, during the transformation of AlaPhe to AlaAla, both the phenyl ring and methyl group were attached to the second amino acid residue at the same time. However, branching was done at the C_β atom rather than C_α to minimise the number of alchemical transformations. All transformations involved only the non-bonded interactions while bonded interactions were kept the same throughout the simulations (Boresch and Karplus, 1999; Boresch, 2002). Two sets of MD simulations were run using GROMACS 4.5.4 (Hess et al., 2008): (i) the transformation of peptides bound to PepT_{St} (bound simulation) and (ii) the transformation of peptides in solution (unbound). Each transformation was divided into 3 steps: (i) removing the

partial charges of the disappearing chemical groups, (ii) removing the van de Waals interactions of the disappearing groups while adding that of the emerging groups, and (iii) adding the partial charges of the emerging groups. The transformation was done by coupling the non-bonded potential energy terms to a scaling parameter λ, where at λ = 0, the non-bonded terms of AlaPhe were used whilst at λ = 1, the non-bonded terms of the final peptide were used. A soft core potential (Beutler et al., 1994) was applied in step (ii) to avoid singularities and instabilities. For each step, we performed 11 independent 5 ns MD simulations at λ = 0, 0.1, 0.2, 0.3, 0.4, 0.5, 0.6, 0.7, 0.8, 0.9, 1. The $\partial U/\partial\lambda$ values were computed every 1 ps and extracted using GROMACS `g_energy` tool. These were integrated for all λ values using the trapezoid rule along the simulation trajectory to obtain values of ΔG.

For our prediction steps (Figure 3C), a model of PepT_{St}-AlaAla was used for the starting coordinates and the alanine side chain on either the N- or C-terminus was transmuted to phenylalanine, aspartate, glutamate or lysine. For transformations involving a change in the total charge of the peptide substrate, for example from AlaAla to AlaAsp, both the bound and unbound simulations were performed in the same simulation box as per described in Rashid et al. (2013)–while the AlaAla to AlaAsp transformation was applied to PepT_{St} in the binding site, the reverse transformation (AspAla to AlaAla) was conducted in bulk solution simultaneously to maintain the overall net charge of the system. For predictions with the alternative binding model of AlaLys and LysAla (Figure 5C), the crystal structure of PepT_{St}-triAla was used to model the binding of AlaAla in either the Bottom or the Top models, which is then used as the starting coordinates. Again, either the N- or C-terminus of this AlaAla dipeptide is transformed to lysine.

Equilibration and convergence times for each simulation was estimated based on the reverse cumulative averaging method (Yang et al., 2004). Only de-correlated energy outputs from the equilibrated period of the simulations were used in the calculation of ΔG. For our validation step, the performance of each prediction method was assessed by the Spearman's rank correlation coefficient (Lehmann and D'Abrera, 1998) following the equation below:

$$\rho = 1 - \frac{6\sum(x_i - y_i)^2}{n(n^2-1)}$$

where x_i is the ranking for experimental IC_{50} values, y_i is the ranking for predicted ΔG values and n is the size of the dataset.

2.4 Competition transport assays

Proton-driven competition uptake assays were performed as described in Solcan et al. (2012). Proteoliposomes (with 5 μg $PepT_{St}$) in internal buffer (20 mM Potassium Phosphate, 100 mM Potassium Acetate, 2 mM Magnesium Sulphate, pH 6.5) were diluted into external buffer (120 mM Sodium Phosphate, 2 mM Magnesium Sulphate, pH 6.5) containing 25 μM 3H labelled AlaAla (reporter substrate) in the presence of increasing peptide concentration (substrate of interest). Reaction was initiated through the addition of 10 μM valinomycin and terminated after 4 minutes by dilution into 0.1 M Lithium Chloride and collected on nitrocellulose filters prior to scintillation counting. The 3H signal was converted to molar concentrations of peptide using standard curves for each substrate.

References

Aqvist, J., Medina, C., and Samuelsson, J. (1994). A new method for predicting binding affinity in computer-aided drug design. Protein Eng. *73*, 385–91.

Beutler, T., Mark, A., and van Schaik, R. (1994). Avoiding singularities and numerical instabilities in free energy calculations based on molecular simulations. Chem. Phys. Lett. *222*, 529–539.

Boresch, S. (2002). The Role of Bonded Energy Terms in Free Energy Simulations - Insights from Analytical Results. Mol. Sim. *28*, 13–37.

Boresch, S. and Karplus, M. (1999). The Role of Bonded Terms in Free Energy Simulations: 1. Theoretical Analysis. J. Phys. Chem. A *103*, 103–118.

Bussi, G., Donadio, D., and Parrinello, M. (2007). Canonical sampling through velocity rescaling. J. Chem. Phys. *126*, 014101.

Cheng, T., Li, X., Li, Y., Liu, Z., and Wang, R. (2009). Comparative assessment of scoring functions on a diverse test set. J. Chem. Inf. Model. *49*, 1079–93.

Dickson, C.J., Rosso, L., Betz, R.M., Walker, R.C., and Gould, I.R. (2012). GAFFlipid: a General Amber Force Field for the accurate molecular dynamics simulation of phospholipid. Soft Matter *8*, 9617.

Essmann, U., Perera, L., Berkowitz, M.L., Darden, T., Lee, H., and Pedersen, L.G. (1995). A smooth particle mesh Ewald method. J. Chem. Phys. *103*, 8577.

Hansson, T., Marelius, J., and Aqvist, J. (1998). Ligand binding affinity prediction by linear interaction energy methods. J. Comput. Aided Mol. Des. *12*, 27–35.

Hess, B., Bekker, H., Berendsen, H.J.C., and Fraaije, J.G.E.M. (1997). LINCS: A linear constraint solver for molecular simulations. J. Comp. Chem. *18*, 1463–1472.

Hess, B., Kutzner, C., and Spoel, D.V.D. (2008). GROMACS 4: Algorithms for highly efficient, load-balanced, and scalable molecular simulation. J. Chem. Theory Comput. *4*, 435–447.

Hou, T., Wang, J., Li, Y., and Wang, W. (2011). Assessing the performance of the MM/PBSA and MM/GBSA methods. 1. The accuracy of binding free energy calculations based on molecular dynamics simulations. J. Chem. Inf. Model. *51*, 69–82.

Humphrey, W. and Dalke, A. (1996). VMD: visual molecular dynamics. J. Mol. Graph. *15*, 33–38.

Jorgensen, W.L., Chandrasekhar, J., Madura, J.D., Impey, R.W., and Klein, M.L. (1983). Comparison of simple potential functions for simulating liquid water. J. Chem. Phys. *79*, 926.

Lehmann, E.L. and D'Abrera, H.J.M. (1998). Nonparametrics: Statistical Methods Based on Ranks, Revised (Englewood Cliffs, NJ: Prentice-Hall).

Lindorff-Larsen, K., Piana, S., Palmo, K., Maragakis, P., Klepeis, J.L., Dror, R.O., and Shaw, D.E. (2010). Improved side-chain torsion potentials for the Amber ff99SB protein force field. Proteins *78*, 1950–8.

Luzhkov, V.B. and Aqvist, J. (2001). Mechanisms of tetraethylammonium ion block in the KcsA potassium channel. FEBS Lett. *495*, 191–6.

Lyons, J.A., Parker, J.L., Solcan, N., Brinth, A., Li, D., Shah, S.T., Caffrey, M., and Newstead, S. (2014). Structural basis for polyspecificity in the POT family of proton-coupled oligopeptide transporters. EMBO Rep. 1–8.

Miller III, B., Jr, T.M., Swails, J.M., Homeyer, N., Gohlke, H., and Roitberg, A.E. (2012). MMPBSA.py : An Efficient Program for End-State Free Energy Calculations. J. Chem. Theory Comput. *8*, 3314–3321.

Osterberg, F. and Aqvist, J. (2005). Exploring blocker binding to a homology model of the open hERG K+ channel using docking and molecular dynamics methods. FEBS Lett. *579*, 2939–44.

Parrinello, M. (1981). Polymorphic transitions in single crystals: A new molecular dynamics method. J. Appl. Phys. *52*, 7182.

Rashid, M.H., Heinzelmann, G., Huq, R., Tajhya, R.B., Chang, S.C., Chhabra, S., Pennington, M.W., Beeton, C., Norton, R.S., and Kuyucak, S. (2013). A potent and selective peptide blocker of the Kv1.3 channel: Prediction from free-energy simulations and experimental confirmation. PLoS One *8*.

Sali, A. and Blundell, T. (1994). Comparative protein modelling by satisfaction of spatial restraints. J. Mol. Biol. *234*, 779–815.

Shen, M., Devos, D., Melo, F., and Sali, A. (2006). A composite score for predicting errors in protein structure models. Protein Sci. *15*, 1653–1666.

Solcan, N., Kwok, J., Fowler, P.W., Cameron, A.D., Drew, D., Iwata, S., and Newstead, S. (2012). Alternating access mechanism in the POT family of oligopeptide transporters. EMBO J. 1–11.

Trott, O. and Olson, A.J. (2010). Software News and Update AutoDock Vina : Improving the Speed and Accuracy of Docking with a New Scoring Function, Efficient Optimization, and Multithreading. J. Comput. Chem. *31*, 455–461.

Wang, J., Wolf, R.M., Caldwell, J.W., Kollman, P., and Case, D. (2004). Development and testing of a general amber force field. J. Comp. Chem. *25*, 1157–74.

Wolf, M.G., Hoefling, M., Aponte-santamaría, C., Grubmüller, H., and Groenhof, G. (2010). g_membed : Efficient Insertion of a Membrane Protein into an Equilibrated Lipid Bilayer with Minimal Perturbation. J. Comp. Chem. *31*, 2169–2174.

Yang, W., Bitetti-Putzer, R., and Karplus, M. (2004). Free energy simulations: use of reverse cumulative averaging to determine the equilibrated region and the time required for convergence. J. Chem. Phys. *120*, 2618–28.
