# Supplementary material for: Accurate Prediction of Ligand Affinities for a Proton-Dependent Oligopeptide Transporter
Source: Cell Chem Biol. 2016 Feb 18;23(2):299–309. doi: 10.1016/j.chembiol.2015.11.015 (PMC4760754; doi:10.1016/j.chembiol.2015.11.015)
Supplement: Document S2. Article plus Supplemental Information [file mmc2.pdf]

Resource

Cell Chemical Biology

# Accurate Prediction of Ligand Affinities for a Proton-Dependent Oligopeptide Transporter

## Graphical Abstract

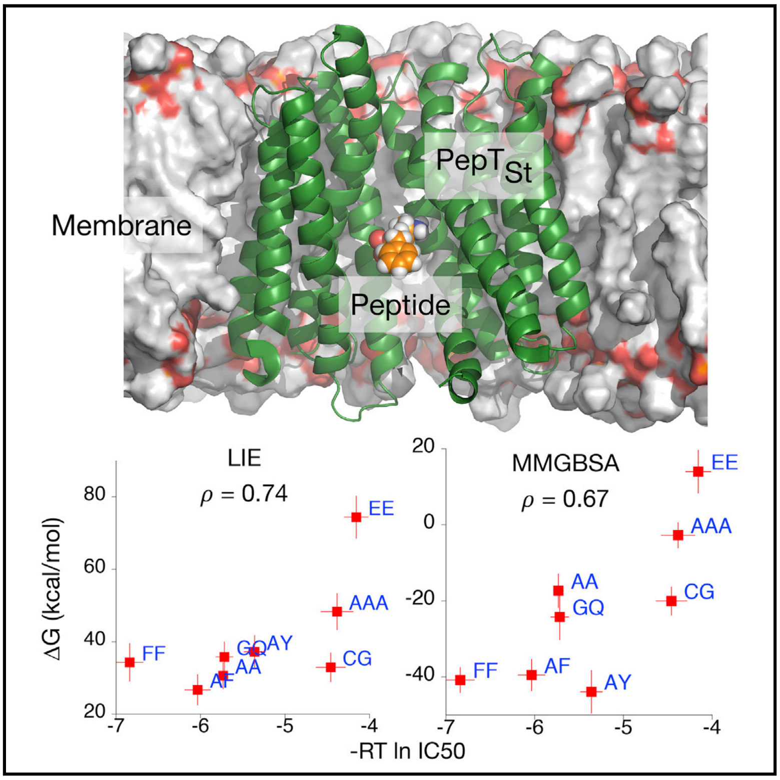


## Highlights

- A hierarchical computational approach determines ligand affinities to transporters
- Lysine-containing dipeptides proposed to bind vertically like a tripeptide
- Experimental structures are vital for the accurate prediction of affinities
- A model of prodrug interactions to human PepT1 is suggested

## Authors

Firdaus Samsudin, Joanne L. Parker, Mark S.P. Sansom, Simon Newstead, Philip W. Fowler

## Correspondence

simon.newstead@bioch.ox.ac.uk (S.N.),
philip.fowler@bioch.ox.ac.uk (P.W.F.)

## In Brief

Samsudin et al. demonstrate how a hierarchical free energy method can predict the affinities of ligands for a peptide transporter protein. This approach may be used to optimize drug-transporter interactions.

Samsudin et al., 2016, Cell Chemical Biology *23*, 299–309
February 18, 2016 ©2016 The Authors
http://dx.doi.org/10.1016/j.chembiol.2015.11.015

CellPress

Please cite this article as: Samsudin et al., Accurate Prediction of Ligand Affinities for a Proton-Dependent Oligopeptide Transporter, Cell Chemical Biology (2016), http://dx.doi.org/10.1016/j.chembiol.2015.11.015

Cell Chemical Biology
Resource

CellPress

# Accurate Prediction of Ligand Affinities for a Proton-Dependent Oligopeptide Transporter

Firdaus Samsudin,[1] Joanne L. Parker,[1] Mark S.P. Sansom,[1] Simon Newstead,[1,*] and Philip W. Fowler[1,*]
[1]Department of Biochemistry, University of Oxford, South Parks Road, Oxford OX1 3QU, UK
*Correspondence: simon.newstead@bioch.ox.ac.uk (S.N.), philip.fowler@bioch.ox.ac.uk (P.W.F.)
http://dx.doi.org/10.1016/j.chembiol.2015.11.015
This is an open access article under the CC BY license (http://creativecommons.org/licenses/by/4.0/).

## SUMMARY

**Membrane transporters are critical modulators of drug pharmacokinetics, efficacy, and safety. One example is the proton-dependent oligopeptide transporter PepT1, also known as SLC15A1, which is responsible for the uptake of the β-lactam antibiotics and various peptide-based prodrugs. In this study, we modeled the binding of various peptides to a bacterial homolog, PepT$_{St}$, and evaluated a range of computational methods for predicting the free energy of binding. Our results show that a hybrid approach (endpoint methods to classify peptides into good and poor binders and a theoretically exact method for refinement) is able to accurately predict affinities, which we validated using proteoliposome transport assays. Applying the method to a homology model of PepT1 suggests that the approach requires a high-quality structure to be accurate. Our study provides a blueprint for extending these computational methodologies to other pharmaceutically important transporter families.**

## INTRODUCTION

The application of computational chemistry in drug development has centered for a long time around indirect ligand-based techniques, such as pharmacophore modeling and 3D-QSAR studies (Caporuscio and Tafi, 2011). Recently, the emergence of X-ray crystal structures of pharmaceutically important membrane proteins has shifted the paradigm toward direct structure-based approaches, for example, computing the free energy of binding of relevant ligands to a protein, and thereby finding or optimizing lead compounds. Scoring functions are currently the method of choice due to their cheap computational cost (Chen and Shoichet, 2009; Schlessinger et al., 2011; Geier et al., 2013). While this method can work for screening a large library of compounds to produce an initial list of candidates, more robust techniques are needed for accurately predicting binding, such as for ranking ligands, especially for highly dynamic proteins like membrane transporters.

Understanding how a drug candidate interacts with membrane transporters is becoming an important step in drug development (Giacomini et al., 2010). Compelling clinical evidence indicates that membrane transporters expressed in the epithelia of the intestine, kidney, and liver, and in the endothelia of the blood-brain barrier influence not only drug absorption and distribution (Dobson and Kell, 2008) but also their therapeutic efficacy and potential adverse reactions (Shitara and Sugiyama, 2002; Cusatis et al., 2006). A recent update of the U.S. Food and Drug Administration guidelines includes extensive recommendations on in vitro and in vivo studies of transporter-mediated drug-drug interactions (U.S. Department of Health and Human Services et al., 2012). Most of the key transporters that have been characterized belong to two major superfamilies: ATP-binding cassette transporters and solute carriers (SLCs). Of particular interest to this paper is the well-studied and pharmacologically important proton-dependent oligopeptide transporters (POT) family member, PepT1, also known as solute carrier family 15 member 1 (SLC15A1), which is the key representative of clinically important SLC transporters involved in drug transport.

PepT1 is expressed predominantly in the intestinal epithelia (Fei et al., 1994; Shen et al., 1999) and plays a crucial role in maintaining nitrogen homeostasis by coupling the uptake of dipeptides and tripeptides to the proton electrochemical gradient (Daniel and Spanier, 2006). Based on the 20 naturally occurring amino acids, there are more than 8,000 possible peptides that could be its substrates, most of which are expected to be transported (Ito et al., 2013). PepT1, therefore, has a highly promiscuous binding site that can accommodate a wide range of ligands with diverse structures and chemistries. In addition to nutritional peptides, it is well established that PepT1 also recognizes and transports a range of drug compounds, such as many β-lactam antibiotics (Luckner and Brandsch, 2005) and the tumor suppressor bestatin (Inui et al., 1992). The promiscuity of this transporter has been exploited in the development of prodrugs such as the antiviral acyclovir (Ganapathy et al., 1998) and the antihypotensive drug midodrine (Tsuda et al., 2006). In both cases, an amino acid residue was attached to the active drug moiety via an esterification reaction, leading to a compound that is transported across the lining of the gut by PepT1, thereby increasing its oral bioavailability. Understanding how ligands interact with these transporters and being able to predict their affinity could, therefore, enable the rational design of drugs with better pharmacokinetics (Brandsch et al., 2008).

Structurally, PepT1 belongs to the major facilitator superfamily (Pao et al., 1998) and so consists of 12 core transmembrane helices divided into N- and C-terminal bundles, with two additional helices observed in the bacterial homologs (Newstead, 2011; Solcan et al., 2012; Doki et al., 2013; Guettou et al., 2013; Zhao et al., 2014; Boggavarapu et al., 2015). The binding site is

Please cite this article as: Samsudin et al., Accurate Prediction of Ligand Affinities for a Proton-Dependent Oligopeptide Transporter, Cell Chemical Biology (2016), http://dx.doi.org/10.1016/j.chembiol.2015.11.015



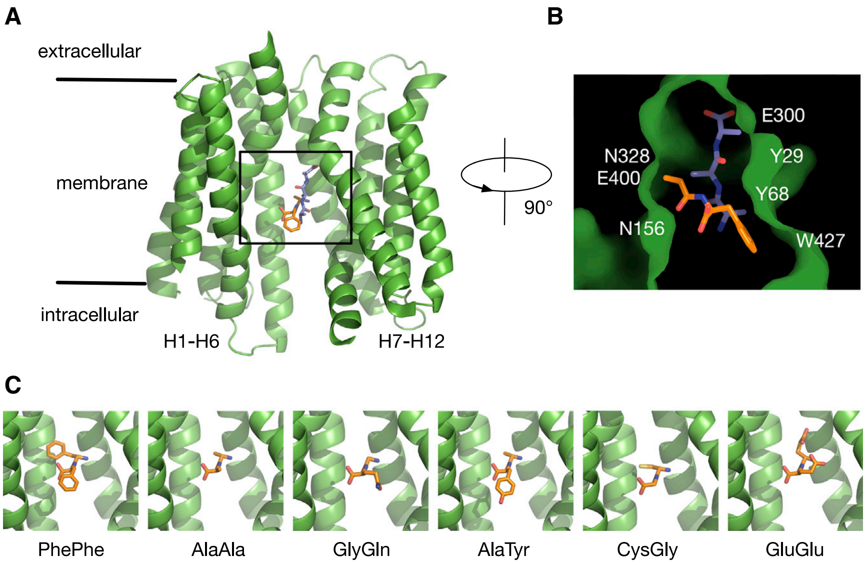


**Figure 1. Modeling the Binding of Peptides Based on the Crystal Complexes of $PepT_{St}$**

(A) Superposition of the two binding modes of peptides to $PepT_{St}$. The dipeptide AlaPhe (orange) binds in a horizontal orientation (PDB: 4D2C), whereas the tripeptide triAla (purple) binds in a vertical orientation (PDB: 4D2D) (Lyons et al., 2014). For clarity, transmembrane helices H2, H11, HA, and HB are removed.

(B) Surface representation of the binding pocket of $PepT_{St}$ with the positions of key interacting residues outlined.

(C) Models of six dipeptides bound to $PepT_{St}$.

positioned between the two bundles and is exposed to either side of the membrane as per the alternating access mechanism (Radestock and Forrest, 2011), with distinct helices controlling this process in a cooperative scissor-like motion (Fowler et al., 2015). The bacterial isoforms share a high sequence identity with mammalian PepT1 within the peptide-binding site, suggesting there may be universally conserved binding and transport mechanisms (Newstead, 2014). Recent high-resolution structures of a homolog from *Streptococcus thermophilus*, $PepT_{St}$, demonstrated that there are at least two binding orientations within this cavity: the dipeptide AlaPhe (PDB: 4D2C) adopted a horizontal pose with respect to the plane of the membrane, whereas the tripeptide AlaAlaAla (triAla, PDB: 4D2D) bound vertically (Lyons et al., 2014), consistent with the dual proton:peptide stoichiometry observed for this transporter (Parker et al., 2014). A second, lower-resolution study resolved the structures of two tripepides (AlaAlaAla, PDB: 4TPJ, and brominated AlaTyrAla, PDB: 4TPG) and a dipeptide (brominated AlaTyr, PDB: 4TPH) when bound to $PepT_{So2}$ (Guettou et al., 2014). For this protein, all three peptides bound in the same horizontal pose with respect to the membrane. These studies have provided essential insights into the molecular basis of promiscuity by the POT transporters, but also raise a further question of how the numerous other di- and tripeptides and, more importantly, drugs interact?

In this article, we develop a computational approach for accurately predicting the affinities of ligands to the peptide transporters. We modeled the binding of various dipeptides based on the crystal structure of the $PepT_{St}$-AlaPhe complex, and used a range of in silico free energy methods to predict their binding affinities. All these methods are very well established and have been applied to a wide range of protein-ligand systems (Chodera et al., 2011). We find that the moderately cheap endpoint methods provide a fast way to classify these ligands into good and poor binders. Our results suggest $PepT_{St}$ generally prefers neutral over charged substrates. Applying a more rigorous theoretical method to a series of dipeptides reveals the importance of the N-terminal side chain in determining the overall affinity. Using a proton-driven competition uptake assay, we validated these predictions but found discrepancies with basic dipeptides. We suggest that these dipeptides bind in a vertical orientation, similar to the previously observed triAla $PepT_{St}$ complex, to accommodate their large side chains. Applying the method to a homology model of human PepT1 suggests that the accuracy of this method depends on the quality of the available protein structure. Nevertheless, our results help to explain how the prodrug approach has worked for PepT1 by revealing the importance of the N- and C-terminal interactions in the binding site. Overall, this study demonstrates how in silico methodologies can work in tandem with in vitro assays to predict ligand affinities in a pharmaceutically relevant membrane transporter.

## RESULTS

### Endpoint and Exact Free Energy Methods Accurately Predict Affinities

To determine which computational methods can best predict the binding of di- and tripeptides to $PepT_{St}$, we needed a test set of peptides and a selection of computational methods to validate. For the test set, we chose seven dipeptides and one tripeptide (triAla) for which experimental transport data were available. Crystal structures of one dipeptide (AlaPhe) and one tripeptide (triAla) bound to $PepT_{St}$ are known (PDB: 4D2C, 4D2D, respectively; Lyons et al., 2014). The pose of the other six dipeptides was assumed to be the same as AlaPhe, as illustrated in Figure 1. We then selected a range of computational methods for calculating binding free energies which we would validate using the test set. The methods can be categorized based on the amount of computational resource each requires (Figure 2D); at the low end is the structure-based scoring function found in AutoDock Vina (Trott and Olson, 2010). Next we chose three different endpoint methods: the linear interaction energy (LIE; Aqvist et al., 1994), molecular mechanics generalized Born surface area (MMGBSA; Onufriev et al., 2000), and molecular mechanics Poisson Boltzmann surface area (MMPBSA; Kollman et al., 2000). All of these require some molecular dynamics (MD) simulation and hence are more expensive. Finally, we selected a theoretically exact method, thermodynamic integration (TI; Kirkwood, 1935) to calculate differences in binding free energies ($\Delta\Delta G$) to refine the other predictions. Experimental binding data for $PepT_{St}$, and POT transporters in general remain scarce,

Please cite this article as: Samsudin et al., Accurate Prediction of Ligand Affinities for a Proton-Dependent Oligopeptide Transporter, Cell Chemical Biology (2016), http://dx.doi.org/10.1016/j.chembiol.2015.11.015



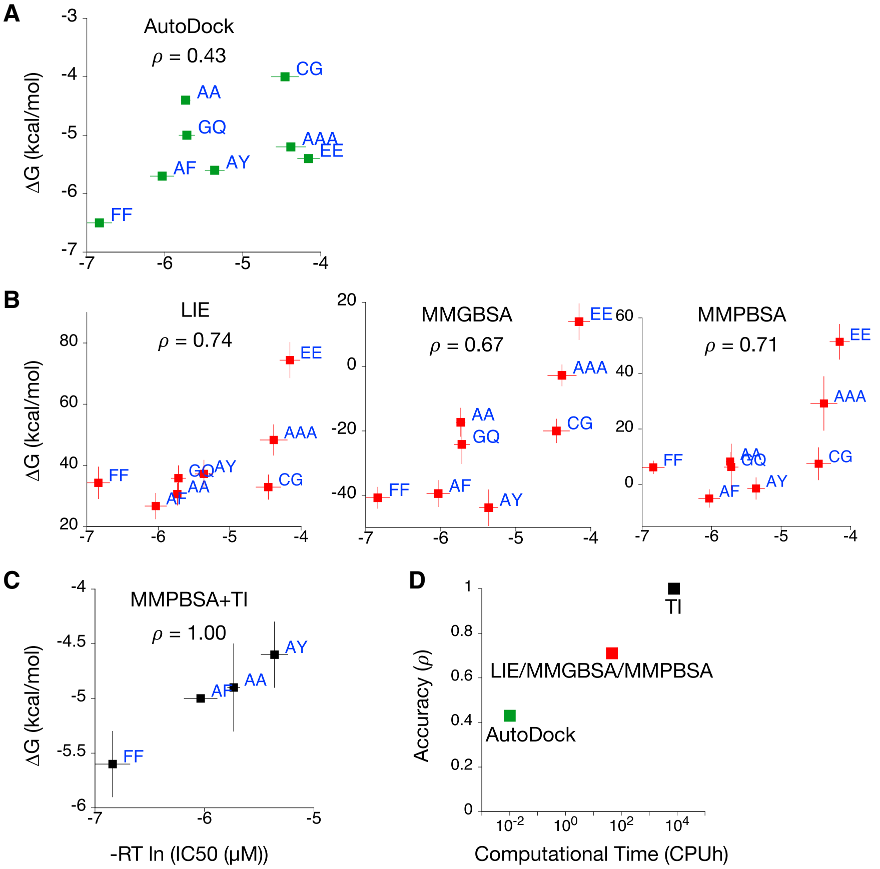


**Figure 2. Endpoint and Exact Free Energy Methods can Predict Affinities Accurately**

(A–C) Predicted ΔG from free energy methods: (A) scoring functions (fast method), (B) endpoint methods (intermediate methods), and (C) theoretically exact method (slow method), compared with $IC_{50}$ values from transport assay. The magnitudes of ΔG obtained from endpoint methods are significantly larger than expected due to the absence of an entropic term in the calculation. Spearman's correlation coefficient, ρ, was calculated to measure the ability of each method to reproduce the same ranking as experimental data. Y error bars indicate statistical errors from de-correlated and equilibrated ΔG data during MD simulations, while X error bars indicate the standard deviations from triplicate experiments.

(D) The performance of all prediction methods and the computational cost based on a quad core processor. AutoDock does not require any simulation and each docking protocol takes around 30 s, and therefore is plotted as $10^{-2}$ CPUh. Standard one-letter code abbreviations have been used for all di- and tri-peptides.

and the standard method for estimating affinities is to perform competition transport assays and measure the half maximal inhibitory concentration ($IC_{50}$) values (Solcan et al., 2012). Unlike certain enzymes, however, transporters have more complicated kinetics such that the relationship between $IC_{50}$ and ΔG is unclear (Eraly, 2008). We therefore compared these two datasets in a qualitative manner using Spearman's correlation coefficient (Lehmann and D'Abrera, 1998), ρ, which assesses the ability of each computational approach to reproduce the ranking of substrates based on experimental $IC_{50}$ values.

Since it does not require any MD simulations, the scoring function is the fastest method to estimate binding affinities. Our results with AutoDock (Figure 2A), however, shows that it is also the least accurate (ρ = 0.43). This is not surprising as AutoDock uses a simplified scoring function (Wang et al., 2002). Although not tested in this study, it is possible that other scoring functions may produce better predictions for peptide transporters as no single docking program performs best across all protein families (Warren et al., 2006; Ross et al., 2013). Also, AutoDock does not account for the conformational sampling of the ligand and the residues in the binding site of the protein as it uses only one snapshot of the protein-ligand complex for its calculation. It is worth noting that this may be improved by using multiple conformers of the complex, for example from MD simulations, as has been done with several other membrane transporters (Schlessinger et al., 2011; Geier et al., 2013). As the binding of the peptide test set was modeled using the same structure, AutoDock Vina predicted that they all have very similar ΔG values. For each dipeptide, the range of ΔG values predicted for the nine poses generated is small (~0.5 kcal/mol), although the score for the pose most similar to the crystal structure or homology model is not always the highest (Figure S1). We therefore conclude that AutoDock Vina does not accurately predict peptide-binding affinities for $PepT_{St}$.

Encouragingly, all three endpoint free energy methods managed to rank the peptide test set well (Figure 2B) compared with the experimental data (ρ ≈ 0.7). The predicted ΔG values for the eight peptides span a wider range, allowing us to better distinguish the subset of well-transported peptides (PhePhe, AlaPhe, AlaAla, and AlaTyr) from poorly transported peptides (triAla and GluGlu). We assume that this increase in accuracy is primarily a result of using an ensemble of conformations generated during the MD simulation, which accounts for the conformational sampling of the ligand and the protein. As endpoint methods require only simulations of the bound and unbound states, the computational cost required for each calculation is relatively modest and therefore they are suitable candidates for a high-throughput workflow to differentiate between high-from low-affinity peptides.

Upon closer examination, however, we found that the endpoint methods poorly ranked peptides with similar $IC_{50}$ values, for example the ρ value of the MMPBSA methods for hydrophobic dipeptides with $IC_{50} \leq 100$ µM is 0.0, i.e., random (Figure S2). We hypothesized that the more rigorous method, TI might improve the ranking of AlaPhe, AlaAla, AlaTyr, and PhePhe by calculating the change in ΔG (ΔΔG) when the amino acids in AlaAla were mutated into either Phe or Tyr. These values were subsequently added to the results of the endpoint methods. We found that by implementing this step, we managed

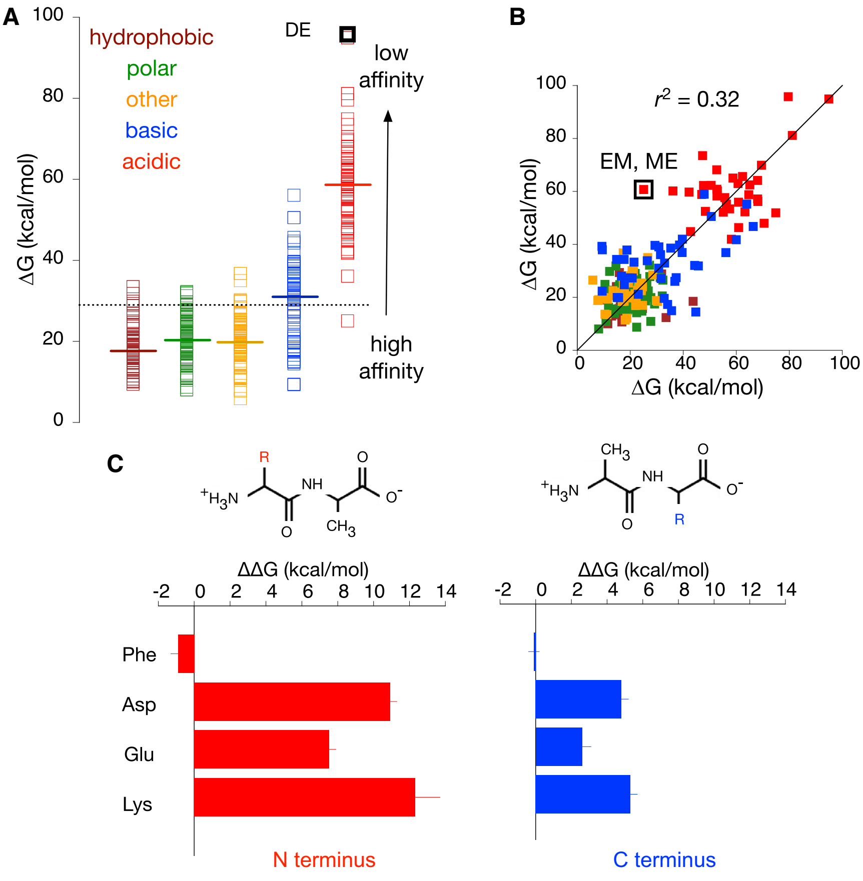


**Figure 3. Further Predictions Suggest that the N-Terminal Side Chain Contributes More Toward the Binding of a Dipeptide than the C Terminus**

(A) ΔG values for all 400 dipeptides as predicted by the LIE method, arranged according to the overall chemical property of the peptide. Solid lines indicate the average of ΔG for each category and the dotted line represents the overall mean ΔG value. The magnitudes of ΔG for all peptides were predicted to be above zero due to the lack of entropic term in the calculation.

(B) ΔG values plotted in pair, each for two dipeptides made of the same residues but in different orders, e.g., GluMet and MetGlu. The line drawn on the graph represents perfect linear correlation.

(C) The TI method was used to calculate ΔΔG of alchemically changing the side chain of either the N or C terminus of AlaAla into Phe, Asp, Glu, or Lys. Errors were calculated by dividing all simulations into an equal number of independent bins as indicated by the reverse cumulative averaging method (Yang et al., 2004).

to significantly improve the prediction and reproduce the exact experimental ranking (ρ = 1.0) (Figure 2C). It is acknowledged, however, that due to the few data points, the apparently higher correlation to experimental data may be artificial.

To quantify and compare the exact amount of resources required for each prediction method, computational usage in hours of single CPU usage (CPUh) was estimated based on the performance of GROMACS for MD simulation using an Intel quad core Xeon processor (Figure 2D). It is no surprise that the more computational input fed into the methods, the more accurate the predictions become. The endpoint methods are an excellent compromise between good performance and low cost. We therefore conclude that it is most efficient to adopt a hybrid approach: using endpoint methods to broadly classify the ligands into high- and low-affinity substrates and then applying TI where necessary to further refine specific predictions.

## Binding of Dipeptides Sensitive to their N-Terminal Residue

Having determined, using the test set of peptides, which free energy methods are most accurate, we now make some predictions for the bacterial transporter $PepT_{St}$ and test them experimentally. Since the LIE method ranked the test set the best, we used it to predict ΔG for all possible 400 dipeptides. We again assumed that all dipeptides bind in the same orientation as AlaPhe, as has been elucidated by Lyons et al. (2014). In agreement with previous studies on PepT1 (Vig et al., 2006), PepT2 (Biegel et al., 2006), and a peptide transporter from *Saccharomyces cerevisiae*, Ptr2p (Ito et al., 2013), our results predict that neutral dipeptides made of hydrophobic and polar amino acids are the preferred substrates for $PepT_{St}$ with almost all binding free energies below average (Figure 3A). As expected, we also found that acidic dipeptides bind least well with the lowest affinities predicted if both side chains are negatively charged, e.g., AspGlu. However, our results suggest that dipeptides with basic residues have moderate affinities for $PepT_{St}$, while an experimental transport assay showed that LysLys has a high $IC_{50}$ value, indicating poor transport (Solcan et al., 2012).

Previous modeling of PepT1 suggested that the binding site predominantly recognizes the peptide backbone and therefore substrate affinities should not be significantly affected by the sequence of residues (Foley et al., 2010). To check if our results agreed with this prediction, we replotted each ΔG value against its sequence-reversed equivalent, i.e., Ala-X versus X-Ala (Figures 3B and S3). If order does not matter, then the points should all fall on a straight line; however, we found a relatively low correlation ($r^2$ = 0.32) with some pairs differing significantly, for example, AlaLys and LysAla. Our results therefore suggest that the order of amino acid residues does influence the overall affinity in $PepT_{St}$.

To explore this further, TI calculations were performed to determine how the binding free energy changes when the N- or C-terminal side chain of AlaAla is transmuted into either phenylalanine, aspartate, glutamate, or lysine (Figure 3C). Negative ΔΔG values (and therefore better binding) were obtained when either side chain was substituted with Phe, exemplifying the inclination of this transporter toward hydrophobic peptides as shown by other POTs (Gebauer and Hartrodt, 2003; Biegel

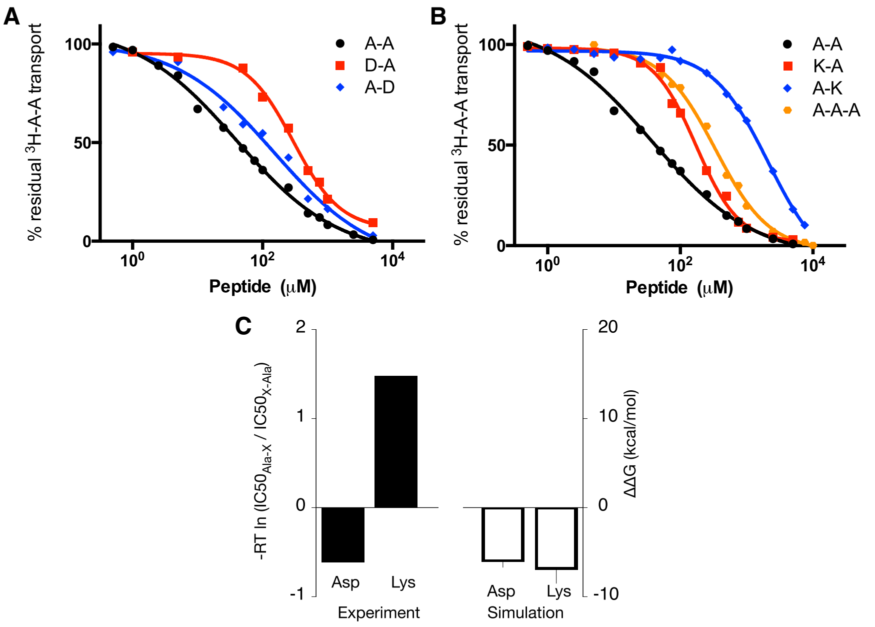


**Figure 4. Proton-Driven Competition Uptake Assays**

(A) $IC_{50}$ competition curves for AlaAsp and AspAla showing residual uptake of $^3$H-AlaAla in a proteoliposome-based transport assay.

(B) Similar $IC_{50}$ competition curves for AlaLys, LysAla, and triAla.

(C) Apparent "ΔΔG" values derived based on the ratio of $IC_{50}$ of Ala-X and X-Ala, compared with the corresponding calculated ΔΔG values from simulation.

Errors were calculated as per Figure 3.

et al., 2006; Doki et al., 2013; Fang et al., 2000). Transformation into a charged residue, however, resulted in a reduction of binding affinity (positive ΔΔG), in agreement with our LIE predictions. In all cases, when the N terminus was altered, the magnitude of the change in binding affinity was larger than the identical change at the C terminus. These results indicate that the N-terminal residue is crucial in determining the selectivity of $PepT_{St}$.

We then investigated which residues in the binding site of the transporter interact with the side chains of the dipeptides (Figure S4). The C-terminal side chain mainly occupies a hydrophobic pocket and forms hydrophobic interactions primarily with Tyr68 and Trp296. The N-terminal side chain, however, contacts a group of polar residues including Tyr30, Asn156, and Asn328. All of these residues have been previously shown to be important for peptide transport by $PepT_{St}$ (Solcan et al., 2012). These analyses suggest that the N-terminal side chain potentially binds more strongly to the binding site via multiple electrostatic interactions and hydrogen bonds, and thereby is more sensitive to changes, whereas the C-terminal counterpart is accommodated inside a hydrophobic pocket that can adapt to various chemical groups without a large energetic penalty, and therefore is less sensitive when perturbed to another side chain.

To test these predictions, we performed proton-driven competition uptake assays for AlaAsp, AspAla, AlaLys, and LysAla using radiolabeled AlaAla as the reporter substrate. The results were then compared with the uptake of the neutral peptides, AlaAla and triAla (Figures 4A and 4B). To make comparison with our in silico predictions easier, we determined the ratio of $IC_{50}$ values between AspAla and AlaAsp as well as between LysAla and AlaLys, and calculated the apparent "ΔΔG" values (Figure 4C). Consistent with our in silico predictions, having the aspartate residue at the N terminus is more detrimental to transport compared with having the same residue at the C terminus, as exemplified by the larger $IC_{50}$ of AspAla (300 μM) compared with AlaAsp (100 μM) and therefore the negative apparent "ΔΔG" value. A similar experiment for positively charged dipeptides, AlaLys and LysAla, however, showed the opposite trend. LysAla has a lower $IC_{50}$ than AlaLys, i.e., 150 μM and 2.1 mM, respectively, and their apparent "ΔΔG" value is therefore positive. This indicates that for lysine, positioning this residue on the C terminus of a dipeptide is more detrimental to its affinity.

## Lysine-Containing Dipeptides are Predicted to Bind in a Tripeptide-like Pose

The discrepancy between our computational predictions and experimental data for positively charged dipeptides could be as a result of many different factors such as inadequate sampling and force field errors. One other possible reason is that these peptides do not bind in the same horizontal pose as AlaPhe. As the vertical pose is the only alternative binding mode observed in crystal structures (Figure 1B), we explored this hypothesis by modeling the binding of AlaAla, AlaLys, and LysAla based on the crystal structure of $PepT_{St}$ bound to triAla (PDB: 4D2D; Lyons et al., 2014). TriAla has three residues, so there are two possible ways of modeling a dipeptide: (1) removing the C-terminal residue and using the first and second residues as the template or (2) removing the N-terminal residue and using the second and third residues as the template. The former approach positions the model dipeptide closer to the cytoplasmic side, and henceforth is called the bottom model, whereas the latter places the peptide nearer to the extracellular side (top model) (Figure 5A). As before, we performed TI to alchemically convert either the N- or C-terminal side chain of AlaAla in this vertical binding modes into lysine, and subsequently calculated how the difference in binding free energy (ΔΔG) between LysAla and AlaLys.

Our results for both the bottom and top models suggest that AlaLys has a less negative ΔG value and thereby binds less tightly compared with LysAla, as suggested by the positive ΔΔG (Figure 5B), in agreement with previously performed transport assays. Further inspection of the alternative binding models suggests that the strong binding of LysAla stems from favorable salt bridges formed via the ε-amino group with Tyr68 and Glu300 in the bottom model (Figure S5A) or Asn156 in the top model (Figure S5B). In contrast, the lysine side chain of AlaLys protrudes

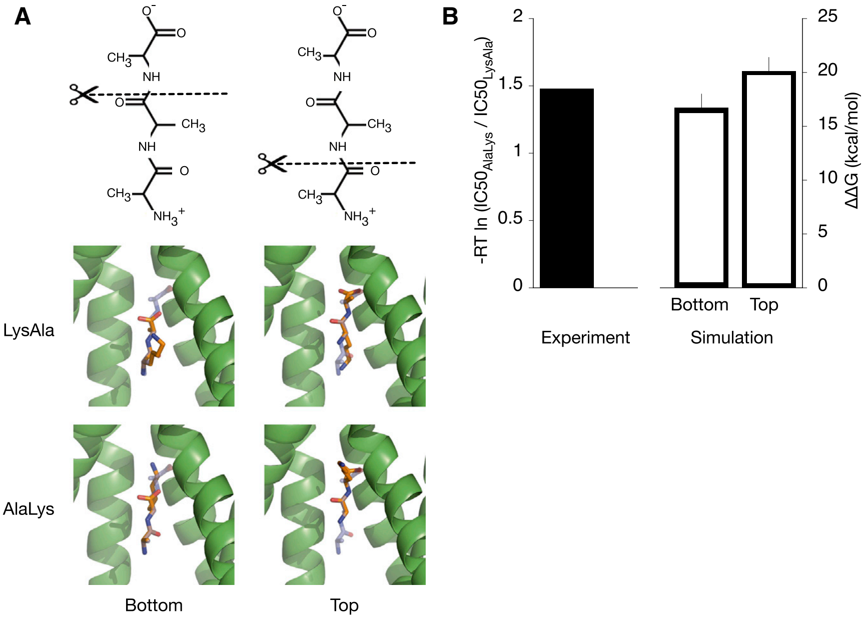


**Figure 5. Re-Modelling the Binding of LysAla and AlaLys Based on a Tripeptide, triAla**

(A) Two ways to model the binding of a dipeptide based on triAla: (1) by removing the C-terminal residue, and using the first and second residues as template (bottom models) and (2) by removing the N-terminal residue, and using the second and third residues as template (top). The figures overlay the new models of AlaLys and LysAla (orange stick representation) on top of triAla (purple).

(B) The TI method was used to calculate ΔΔG of alchemically transforming the N- or C-terminal side chain of AlaAla (in both bottom and top models) into lysine. These are compared with the apparent "ΔΔG" values derived based on the ratio of $IC_{50}$ of AlaLys and LysAla from the transport assays. Errors were calculated as per Figure 3.

into a cavity that is in close proximity to Arg33, therefore resulting in unfavorable electrostatic repulsion and hence a more positive ΔG value (Figure S5D).

While our modeling appears to suggest that two possible vertical binding sites exist for a dipeptide, i.e., the bottom and top models, we acknowledge that in reality this might not be the case. The binding of triAla to $PepT_{St}$ in the original crystal structure showed fewer contacts with residues in the binding cavity compared with AlaPhe, indicating a weak interaction, consistent with the higher $IC_{50}$ value (Lyons et al., 2014). Similarly, in our simulation, a ligand bound in this orientation was able to move up and down, suggesting that there are no distinct "bottom" and "top" binding sites, instead there is just one large vertical cavity. Hence, one would expect to obtain similar values of ΔΔG for both models. That this is not the case suggests the TI simulations have not converged, which is not surprising given the likely timescale for the ligand to explore the vertical pocket. Despite this, as they both show the same trend as experiment, our current results suggest that AlaLys and LysAla bind in a similar orientation to triAla, rather than the horizontal pose of AlaPhe. Nevertheless, further high-resolution crystal structures are still required to verify the validity of our prediction for these peptides.

## Experimental Structures are Essential for Accurate Predictions

A primary goal of this study is to develop a transferable approach of relevance to drug discovery. We therefore tried our method on a homology model of the pharmaceutically relevant human PepT1 (Beale et al., 2015) and expanded the test set to include 14 drug compounds that are known substrates of this transporter. Peptides were assumed to bind to PepT1 in the same way as to $PepT_{St}$. The binding of drugs was modeled based on the conformations of either AlaPhe or triAla according to their size. Our predicted ΔG values were then compared with the $IC_{50}$ data from whole-cell transport assays (Biegel et al., 2005; Vig et al., 2006). Unfortunately, all methods failed to distinguish between good and poor binders with correlation coefficients of ~0.0 (indicating that the predictions are random) (Figure S6).

One potential reason for this loss of predictive ability is the lack of a high-resolution crystal structure of human PepT1. To investigate how much the approach depends on the quality of the protein structure, a series of homology models for the bacterial homolog $PepT_{St}$ were built using as templates, in decreasing order of quality: (1) the crystal structure of $PepT_{St}$ itself (PDB: 4D2C and 4D2D), (2) the crystal structure of GkPOT (PDB: 4IKV) that shares ~50% sequence identity with $PepT_{St}$, and (3) the crystal structure of LacY (PDB: 1PV6), which has only ~25% sequence homology compared with $PepT_{St}$. For the same peptide test set as before, all three homology models showed lower correlations for all methods compared with predictions using the crystal structure of $PepT_{St}$. Interestingly, the predictive ability of all the endpoint methods degrades proportionally to the quality of the protein structure (Figure 6), with the best model showing a slight decrease in the ρ value, followed by larger decreases for the intermediate and poor-quality models. Taken together, these results suggest that, while our current approach can be applied to other members of the peptide transporter family, a high-resolution crystal or electron microscopy structure is essential to achieve accurate results.

## DISCUSSION

We have demonstrated that computational modeling, molecular simulation, and free energy calculations can accurately predict protein-ligand interactions for a membrane transporter. Using an endpoint method, LIE, we first classified all 400 possible dipeptide ligands into strong and weak binders. In agreement with experimental data from other peptide transporters (Vig et al., 2006; Biegel et al., 2006; Ito et al., 2013), we found that uncharged peptides are, on average, the best substrates, while acidic residues bind weakly. Refining these results using a theoretically rigorous method, TI, we then found that the N-terminal residue of a dipeptide contributes significantly toward selectivity. These predictions were tested experimentally, which

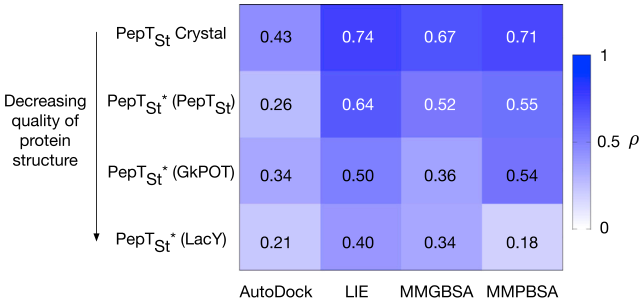


**Figure 6. Comparison of Performance of Binding Affinity Predictions Using a Scoring Function (AutoDock) and Endpoint Methods, LIE, MMGBSA, and MMPBSA, for Homology Models of PepT$_{St}$ as Marked with Asterisks**

The template used for each model is shown in parentheses. The number in each box represents Spearman's correlation coefficient, ρ, between predicted ΔG and experimental $IC_{50}$ data for a test set of eight peptides (as shown in Figure 2). These are colored from white (0.0) to blue (1.0).

showed good agreement except for basic dipeptides. Remodeling the basic peptides based on the vertical binding orientation restored agreement between simulation and experiment, therefore we hypothesize that these basic dipeptides may not interact with PepT$_{St}$ in the canonical dipeptide-binding pose, but instead possibly mimic a tripeptide.

It is important at this stage to highlight the limitations of our computational approach. The MD simulations were performed using a molecular mechanics force field, which is a classical approximation of a more complex quantum mechanical reality at the atomic level. It is well established, however, that this force field is accurate at reproducing many experimental results (Shirts et al., 2003). The magnitude of the absolute ΔG values calculated by the endpoint methods are large and mostly positive, rather than small and negative as one would expect (Figures 2B and 3A). This is due to various assumptions we have made. The MMGBSA and MMPBSA methods neglect the entropic contributions, ΔS, from the protein and ligand, although we expect the change in entropy to be similar since all of the ligands are structurally similar (Oehme et al., 2012). In principle, one could estimate the change in entropy; however, the currently available methods are not precise and return very large standard errors (Kar et al., 2011). For the LIE method, we implemented hydroxyl-based scaling factors (Hansson et al., 1998) for simplicity, instead of the more sophisticated parametrizations that take into account other chemical groups (Wang et al., 1999; Almlof et al., 2007). While these computed ΔG values should not be considered as the true binding free energy, they give meaningful results when used qualitatively, for example, in the comparison between various ligands to understand the general trend of binding affinity. Our modeling is also limited to the inward open conformation, so the ΔG calculated might not be representative for other conformations of the transporter. Despite these limitations, our predictions for PepT$_{St}$ using the peptide test set show good agreement with experimental data, suggesting that these methods are indeed sufficient to study ligand interactions.

A ligand-based substrate template of PepT1 (Bailey et al., 2000) suggested that dipeptides are better substrates than tripeptides. However, this template fails to account for the generally poor transport behavior of basic dipeptides that show worse affinities than neutral tripeptides (Eddy et al., 1995; Terada et al., 2000). Here, we propose based on computer modeling and competition assays that the lower affinities of these dipeptides arise from a different binding pose. Unlike neutral and acidic dipeptides, a dipeptide with lysine residues is proposed to bind like a tripeptide in a vertical binding orientation, which results in poorer transport as this binding mode is less tightly coordinated (Lyons et al., 2014). The large extended lysine side chain makes the total length of AlaLys and LysAla ~10 Å, which is about the same as the backbone of a tripeptide. As such, positioning these dipeptides laterally like AlaPhe may cause unfavorable steric clashes and the only way for them to bind is by treating their large side chain effectively as an additional residue. As the side chain of an arginine residue is of similar length and is also capped by a positive charge, we conjecture that dipeptides with arginine may also interact this way, which would explain their poor uptake.

The importance of the N terminus for peptide binding to PepT1 has been demonstrated by previous studies (Borner et al., 1998; Meredith et al., 2000), which suggested that the amino group is responsible for aligning the rest of the molecule in the central binding cavity. Similar results were observed for Ptr2p (Ito et al., 2013), where the N-terminal residue showed a higher propensity toward determining whether a substrate belongs to a high- or low-affinity group. Our studies lend further support to this idea by showing that the side chain on the N terminus is the primary determinant of binding selectivity in PepT$_{St}$. The structural reasoning behind this observation is that this side chain contacts a group of polar residues in the binding site via multiple electrostatic interactions, while the C-terminal side chain is surrounded by an electro-neutral binding cavity. As PepT$_{St}$ and human PepT1 share 80% sequence identity within the peptide-binding site, it is likely that PepT1 has a similar substrate recognition mechanism. This would explain why the peptide prodrug approach targeting PepT1 has been very successful. These prodrugs are structurally analogous to a dipeptide, and the added amino acid acts as a pseudo N terminus and the active drug moiety the C terminus (Figure 7). While the N-terminal amino acid is crucial to gain affinity to the transporter, the drug compound itself fits well inside the large hydrophobic cavity, and hence the prodrugs are recognized and transported by PepT1. This prodrug recipe has been employed for various drugs such as the antivirals valacyclovir (Ganapathy et al., 1998), valganciclovir (Sugawara et al., 2000), and cidofovir (McKenna et al., 2005).

Generating a substrate-binding model for a membrane transporter presents enormous possibilities for rational drug design. Previous studies of the mammalian PepT1 transporter produced a simplified two-dimensional model (Meredith et al., 2000), followed later by three-dimensional pharmacophores (Biegel et al., 2005; Vig et al., 2006; Foley et al., 2010; Pedretti et al., 2008). A more detailed structure-based model is imperative following the recent crystallographic and thermodynamic evidence that this transporter operates with at least two distinct binding modes (Parker et al., 2014). We made the best use of these data by combining various computational techniques alongside experimental transport assays to suggest

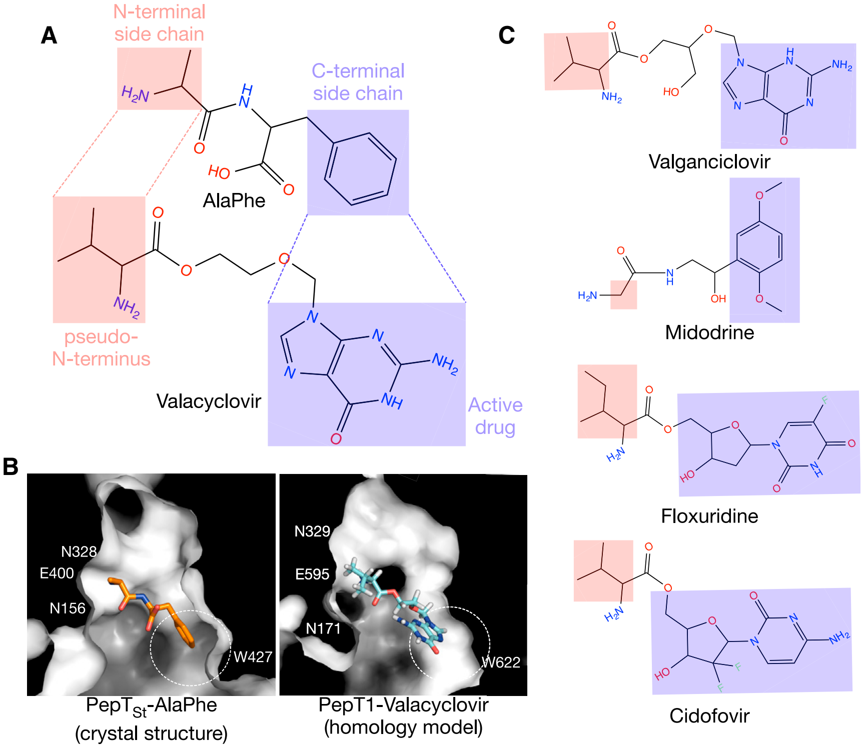


**Figure 7. A Model of Peptide Prodrug Binding to PepT1**

(A) Structural comparison of an antiviral prodrug valacyclovir and AlaPhe suggests that the attached amino acid, valine (red), acts as a pseudo N terminus, whereas the active drug, acyclovir (blue), mimics the position of the second residue of a dipeptide.

(B) Surface representation of the binding site of $PepT_{St}$-AlaPhe (crystal structure) and PepT1-valacyclovir (homology model) highlighting the position of the phenylalanine side chain and acyclovir inside a hydrophobic cavity, depicted by dotted circles.

(C) Other prodrugs that target PepT1 following the approach of adding an amino acid residue to form an N terminus.

how other peptides and drugs interact with this transporter. While it is currently not possible to directly apply our methods to the human PepT1 due to the lack of a high-resolution crystal structure, our promising results with its model system, $PepT_{St}$, demonstrate its potential for the study of drug-transporter interactions. With the ever-increasing computational power and available crystal structures of membrane transporters, we expect this approach to be extended to other relevant transport systems and larger compound libraries in the near future.

## SIGNIFICANCE

**When a drug is taken orally, it has to pass through the lining of the gut to enter the blood circulation and reach its target. Membrane proteins found in the gut epithelium, for example, the human peptide transporter, PepT1, play key roles in mediating the absorption of orally prescribed drugs. Designing drugs that can strongly bind to PepT1 will therefore improve bioavailability, and in turn optimize dosing and minimize undesirable side effects. Here, we demonstrate an accurate way to computationally determine how well a ligand binds to a bacterial homolog of PepT1. This approach is capable of discriminating strong and weak binders and elucidating the origin of ligand selectivity in this transporter, which we then validate using in vitro transport assays. Studying drug-transporter interactions is becoming an important component of rational drug design. We foresee that this method will be more widely employed in the future to study other medically relevant membrane transporter families.**

## EXPERIMENTAL PROCEDURES

The binding of both peptides and drugs was modeled based on the structure of $PepT_{St}$-AlaPhe (PDB: 4D2C) and $PepT_{St}$-triAla (PDB: 4D2D) complexes (Lyons et al., 2014). For predicting the binding free energy, $\Delta G$, using a scoring function (AutoDock Vina; Trott and Olson, 2010), each peptide and drug compound was docked to the binding site of the protein and the affinity score from the pose most similar to the crystal structure (for AlaPhe and triAla) or model (for other ligands) was taken as the $\Delta G$ value. For $\Delta G$ predictions using the endpoint methods, 1-ns MD simulations were performed using GROMACS 4.5.4 (Hess et al., 2008) with parameters explained in detail in the Supplemental Information. We performed two simulations for the LIE method: (1) a protein-ligand complex in membrane and (2) the ligand in bulk solution. The GROMACS tool, g_lie, was used to compute $\Delta G$ values with the $\alpha$ scaling constant set to 0.18 (Luzhkov and Aqvist, 2001; Osterberg and Aqvist, 2005) and the $\beta$ scaling constant set to either 0.5, 0.43, 0.37, or 0.33 based on the number of hydroxyl groups on the ligand molecule (Hansson et al., 1998). For MMGBSA and MMPBSA, the single trajectory protocol was used (Hou et al., 2011), where only the simulation of protein-ligand complex was run and the trajectories of unbound protein and free ligand were extracted from it. The calculation of $\Delta G$ was done using the MMPBSA.py program (Miller et al., 2012) with the implicit solvation parameters (saltcon and istrng) set to 0.15 M.

For predictions of $\Delta\Delta G$ by the TI method, alchemical MD simulations were performed using either the $PepT_{St}$-AlaPhe crystal complex or $PepT_{St}$-AlaAla model for the starting coordinates. The dual topology approach was employed where both the vanishing and growing atoms were represented separately. All transformations involved only the non-bonded interactions, while bonded interactions were kept the same throughout the simulations (Boresch and Karplus, 1999; Boresch, 2002). Two set of MD simulations were run: (1) the transformation of peptide ligand bound to $PepT_{St}$ (bound state) and (2) the transformation of peptide ligand in bulk solution (unbound state). The transformation of both states was divided into three steps: (1) removing the partial charges of the disappearing chemical groups, (2) removing the van de Waals interactions of the disappearing groups while adding that of the emerging groups, and (3) adding the partial charges of the emerging groups. A soft core potential (Beutler et al., 1994) was applied in step (2) to avoid singularities and instabilities. These transformations were coupled to a scaling parameter, $\lambda$, where at $\lambda = 0$, the non-bonded terms of the initial peptide were used, while at $\lambda = 1$, the non-bonded terms of the final peptide were used. Eleven independent 5-ns MD simulations were performed at $\lambda$ = 0, 0.1, 0.2, 0.3, 0.4, 0.5, 0.6, 0.7, 0.8, 0.9, 1. The trapezoid rule was used to integrate the $\partial U/\partial\lambda$ values to obtain $\Delta G$. In the transformations with a change in the charge of the ligand, the one-box approach described by Rashid et al. (2013) was employed.

The performance of each prediction method was assessed by Spearman's rank correlation coefficient (Lehmann and D'Abrera, 1998) using the following equation:

$$\rho = 1 - \frac{6\sum (x_i - y_i)^2}{n(n^2 - 1)},$$

where $x_i$ is the ranking for experimental $IC_{50}$ values, $y_i$ is the ranking for predicted $\Delta G$ values and n is the size of the dataset. To validate our computational predictions, proteoliposome-based competition transport assays were performed as described in Solcan et al. (2012).

## SUPPLEMENTAL INFORMATION

Supplemental Information includes Supplemental Experimental Procedures and six figures and can be found with this article online at http://dx.doi.org/10.1016/j.chembiol.2015.11.015.

## AUTHOR CONTRIBUTIONS

F.S., M.S.P.S., and P.W.F. conceived the project, designed experiments, and wrote the manuscript. F.S. performed computational predictions of ligand affinities with assistance from P.W.F. J.L.P. and S.N. performed experimental transport assays. All authors reviewed the paper.

## ACKNOWLEDGMENTS

P.W.F. is grateful to the Wellcome Trust for funding (092970/788Z/10/Z). S.N. is a Wellcome Trust Investigator (102890/Z/13/Z). F.S. is supported by a Yayasan Terengganu Scholarship from the Malaysian Government. We would also like to thank Nicolae Solcan for helpful discussions.

Received: August 28, 2015
Revised: October 22, 2015
Accepted: November 4, 2015
Published: January 28, 2016

## REFERENCES

Almlof, M., Carlsson, J., and Aqvist, J. (2007). Improving the accuracy of the linear interaction energy method for solvation free energies. J. Chem. Theory Comput. *3*, 2162–2175.

Aqvist, J., Medina, C., and Samuelsson, J. (1994). A new method for predicting binding affinity in computer-aided drug design. Protein Eng. *73*, 385–391.

Bailey, P.D., Boyd, C.A., and Bronk, J.R. (2000). How to make drugs orally active: a substrate template for peptide transporter PepT1. Angew. Chem. Int. Ed. Engl. *39*, 505–508.

Beale, J., Parker, J.L., Samsudin, M.F., Barrett, A.L., Senan, A., Bird, L.E., Scott, D., Owens, R.J., Sansom, M.S., Tucker, S.J., et al. (2015). Crystal structures of the extracellular domain from PepT1 and PepT2 provide novel insights into mammalian peptide transport. Structure *23*, 1889–1899.

Beutler, T., Mark, A., and van Schaik, R. (1994). Avoiding singularities and numerical instabilities in free energy calculations based on molecular simulations. Chem. Phys. Lett. *222*, 529–539.

Biegel, A., Gebauer, S., Hartrodt, B., Brandsch, M., Neubert, K., and Thondorf, I. (2005). Three-dimensional quantitative structure-activity relationship analyses of beta-lactam antibiotics and tripeptides as substrates of the mammalian H+/peptide cotransporter PEPT1. J. Med. Chem. *48*, 4410–4419.

Biegel, A., Knütter, I., Hartrodt, B., Gebauer, S., Theis, S., Luckner, P., Kottra, G., Rastetter, M., Zebisch, K., Thondorf, I., et al. (2006). The renal type H+/peptide symporter PEPT2: structure-affinity relationships. Amino Acids *31*, 137–156.

Boggavarapu, R., Jeckelmann, J.M., Harder, D., Ucurum, Z., and Fotiadis, D. (2015). Role of electrostatic interactions for ligand recognition and specificity of peptide transporters. BMC Biol. *13*, 58.

Boresch, S. (2002). The role of bonded energy terms in free energy simulations - insights from analytical results. Mol. Sim. *28*, 13–37.

Boresch, S., and Karplus, M. (1999). The role of bonded terms in free energy simulations: 1. Theoretical analysis. J. Phys. Chem. A *103*, 103–118.

Borner, V., Fei, Y.J., Hartrodt, B., Ganapathy, V., Leibach, F.H., Neubert, K., and Brandsch, M. (1998). Transport of amino acid aryl amides by the intestinal H+/peptide cotransport system, PEPT1. Eur. J. Biochem. *255*, 698–702.

Brandsch, M., Knütter, I., and Bosse-Doenecke, E. (2008). Pharmaceutical and pharmacological importance of peptide transporters. J. Pharm. Pharmacol. *60*, 543–585.

Caporuscio, F., and Tafi, A. (2011). Pharmacophore modelling: a forty year old approach and its modern synergies. Curr. Med. Chem. *18*, 2543–2553.

Chen, Y., and Shoichet, B.K. (2009). Molecular docking and ligand specificity in fragment-based inhibitor discovery. Nat. Chem. Biol. *5*, 358–364.

Chodera, J., Mobley, D., and Shirts, M. (2011). Alchemical free energy methods for drug discovery: progress and challenges. Curr. Opin. Struct. Biol. *21*, 150–160.

Cusatis, G., Gregorc, V., Li, J., Spreafico, A., Ingersoll, R.G., Verweij, J., Ludovini, V., Villa, E., Hidalgo, M., Sparreboom, A., et al. (2006). Pharmacogenetics of ABCG2 and adverse reactions to gefitinib. J. Natl. Cancer Inst. *98*, 1739–1742.

Daniel, H., and Spanier, B. (2006). From bacteria to man: archaic proton-dependent peptide transporters at work. Physiology (Bethesda) *21*, 93–102.

Dobson, P.D., and Kell, D.B. (2008). Carrier-mediated cellular uptake of pharmaceutical drugs: an exception or the rule? Nat. Rev. Drug Discov. *7*, 205–220.

Doki, S., Kato, H.E., Solcan, N., Iwaki, M., Koyama, M., Hattori, M., Iwase, N., Tsukazaki, T., Sugita, Y., Kandori, H., et al. (2013). Structural basis for dynamic mechanism of proton-coupled symport by the peptide transporter POT. Proc. Natl. Acad. Sci. USA *110*, 11343–11348.

Eddy, E.P., Wood, C., Miller, J., Wilson, G., and Hidalgo, I.J. (1995). A comparison of the affinities of dipeptides and antibiotics for the di-/tripeptide transporter in Caco-2 cells. Int. J. Pharm. *115*, 79–86.

Eraly, S.A. (2008). Implications of the alternating access model for organic anion transporter kinetics. J. Membr. Biol. *226*, 35–42.

Fang, G., Konings, W., and Poolman, B. (2000). Kinetics and substrate specificity of membrane-reconstituted peptide transporter DtpT of *Lactococcus lactis*. J. Bacteriol. *182*, 2530–2535.

Fei, Y.J., Kanai, Y., Nussberger, S., and Ganapathy, V. (1994). Expression cloning of a mammalian proton-coupled oligopeptide transporter. Nature *368*, 563–566.

Foley, D., Rajamanickam, J., Bailey, P.D., and Meredith, D. (2010). Bioavailability through PepT1: the role of computer modelling in intelligent drug design. Curr. Comput. Aided Drug Des. *6*, 68–78.

Fowler, P., Orwick-Rydmark, M., Radestock, S., Solcan, N., Dijkman, P., Lyons, J., Kwok, J., Caffrey, M., Watts, A., Forrest, L., and Newstead, S. (2015). Gating topology of the proton-coupled oligopeptide symporters. Structure *23*, 290–301.

Ganapathy, M.E., Huang, W., Wang, H., Ganapathy, V., and Leibach, F.H. (1998). Valacyclovir: a substrate for the intestinal and renal peptide transporters PEPT1 and PEPT2. Biochem. Biophys. Res. Commun. *246*, 470–475.

Gebauer, S., and Hartrodt, B. (2003). Three-dimensional quantitative structure-activity relationship analyses of peptide substrates of the mammalian H+/peptide cotransporter PEPT1. J. Med. Chem. *46*, 5725–5734.

Geier, E.G., Schlessinger, A., Fan, H., Gable, J.E., Irwin, J.J., Sali, A., and Giacomini, K.M. (2013). Structure-based ligand discovery for the large-neutral amino acid transporter 1, LAT-1. Proc. Natl. Acad. Sci. USA *110*, 5480–5485.

Giacomini, K.M., Huang, S., Tweedie, D.J., Benet, L.Z., Brouwer, K.L.R., Chu, X., Dahlin, A., Evers, R., Fischer, V., Hillgren, K.M., et al. (2010). Membrane transporters in drug development. Nat. Rev. Drug Discov. *9*, 215–236.

Guettou, F., Quistgaard, E.M., Trésaugues, L., Moberg, P., Jegerschöld, C., Zhu, L., Jong, A.J.O., Nordlund, P., and Löw, C. (2013). Structural insights into substrate recognition in proton-dependent oligopeptide transporters. EMBO Rep. *14*, 804–810.

Guettou, F., Quistgaard, E.M., Raba, M., Moberg, P., Löw, C., and Nordlund, P. (2014). Selectivity mechanism of a bacterial homolog of the human drug-peptide transporters PepT1 and PepT2. Nat. Struct. Mol. Biol. *21*, 728–731.

Hansson, T., Marelius, J., and Aqvist, J. (1998). Ligand binding affinity prediction by linear interaction energy methods. J. Comput. Aided Mol. Des. *12*, 27–35.

Hess, B., Kutzner, C., and Spoel, D.V.D. (2008). GROMACS 4: algorithms for highly efficient, load-balanced, and scalable molecular simulation. J. Chem. Theory Comput. *4*, 435–447.

Hou, T., Wang, J., Li, Y., and Wang, W. (2011). Assessing the performance of the MM/PBSA and MM/GBSA methods. 1. The accuracy of binding free energy calculations based on molecular dynamics simulations. J. Chem. Inf. Model. *51*, 69–82.

Inui, K., Tomita, Y., Katsura, T., Okano, T., Takano, M., and Hori, R. (1992). H+ coupled active transport of bestatin via the dipeptide transport system in rabbit intestinal brush-border membranes. J. Pharmacol. Exp. Ther. *260*, 482–486.

Ito, K., Hikida, A., Kawai, S., Lan, V.T.T., Motoyama, T., Kitagawa, S., Yoshikawa, Y., Kato, R., and Kawarasaki, Y. (2013). Analysing the substrate multispecificity of a proton-coupled oligopeptide transporter using a dipeptide library. Nat. Commun. *4*, 2502.

Kar, P., Lipowsky, R., and Knecht, V. (2011). Importance of polar solvation for cross-reactivity of antibody and its variants with steroids. J. Phys. Chem. B *115*, 7661–7669.

Kirkwood, J.G. (1935). Statistical mechanics of fluid mixtures. J. Chem. Phys. *3*, 300.

Kollman, P.A., Massova, I., Reyes, C., Kuhn, B., Huo, S., Chong, L., Lee, M., Lee, T., Duan, Y., Wang, W., et al. (2000). Calculating structures and free energies of complex molecules: combining molecular mechanics and continuum models. Acc. Chem. Res. *33*, 889–897.

Lehmann, E.L., and D'Abrera, H.J.M. (1998). Nonparametrics: Statistical Methods Based on Ranks, Revised (Prentice Hall).

Luckner, P., and Brandsch, M. (2005). Interaction of 31 beta-lactam antibiotics with the H+/peptide symporter PEPT2: analysis of affinity constants and comparison with PEPT1. FEBS J. *59*, 17–24.

Luzhkov, V.B., and Aqvist, J. (2001). Mechanisms of tetraethylammonium ion block in the KcsA potassium channel. FEBS Lett. *495*, 191–196.

Lyons, J.A., Parker, J.L., Solcan, N., Brinth, A., Li, D., Shah, S.T., Caffrey, M., and Newstead, S. (2014). Structural basis for polyspecificity in the POT family of proton-coupled oligopeptide transporters. EMBO Rep. *15*, 1–8.

McKenna, C.E., Kashemirov, B.A., Eriksson, U., Amidon, G.L., Kish, P.E., Mitchell, S., Kim, J.S., and Hilfinger, J.M. (2005). Cidofovir peptide conjugates as prodrugs. J. Organomet. Chem. *690*, 2673–2678.

Meredith, D., Temple, C.S., Guha, N., Sword, C.J., Boyd, C.A., Collier, I.D., Morgan, K.M., and Bailey, P.D. (2000). Modified amino acids and peptides as substrates for the intestinal peptide transporter PepT1. Eur. J. Biochem. *267*, 3723–3728.

Miller, B.R., III, McGee, T.D., Swails, J.M., Homeyer, N., Gohlke, H., and Roitberg, A.E. (2012). MMPBSA.py: an efficient program for end-state free energy calculations. J. Chem. Theory Comput. *8*, 3314–3321.

Newstead, S. (2011). Towards a structural understanding of drug and peptide transport within the proton-dependent oligopeptide transporter (POT) family. Biochem. Soc. Trans. *39*, 1353–1358.

Newstead, S. (2014). Molecular insights into proton coupled peptide transport in the PTR family of oligopeptide transporters. Biochim. Biophys. Acta *1850*, 488–499.

Oehme, D.P., Brownlee, R.T.C., and Wilson, D.J.D. (2012). Effect of atomic charge, solvation, entropy, and ligand protonation state on MM-PB(GB)SA binding energies of HIV protease. J. Comput. Chem. *33*, 2566–2580.

Onufriev, A., Bashford, D., and Case, D.A. (2000). Modification of the generalized born model suitable for macromolecules. J. Phys. Chem. *104*, 3712–3720.

Osterberg, F., and Aqvist, J. (2005). Exploring blocker binding to a homology model of the open hERG K+ channel using docking and molecular dynamics methods. FEBS Lett. *579*, 2939–2944.

Pao, S., Paulsen, I., and Saier, M. (1998). Major facilitator superfamily. Microbiol. Mol. Biol. Rev. *62*, 1–34.

Parker, J.L., Mindell, J.A., and Newstead, S. (2014). Thermodynamic evidence for a dual transport mechanism in a POT peptide transporter. Elife *3*, 1–13.

Pedretti, A., Luca, L.D., Marconi, C., Negrisoli, G., Aldini, G., and Vistoli, G. (2008). Modeling of the intestinal peptide transporter hpept1 and analysis of its transport capacities by docking and pharmacophore mapping. ChemMedChem *3*, 1913–1921.

Radestock, S., and Forrest, L.R. (2011). The alternating-access mechanism of MFS transporters arises from inverted-topology repeats. J. Mol. Biol. *407*, 698–715.

Rashid, M.H., Heinzelmann, G., Huq, R., Tajhya, R.B., Chang, S.C., Chhabra, S., Pennington, M.W., Beeton, C., Norton, R.S., and Kuyucak, S. (2013). A potent and selective peptide blocker of the Kv1.3 channel: prediction from free-energy simulations and experimental confirmation. PLoS One *8*, e78712.

Ross, G., Morris, G., and Biggin, P. (2013). One size does not fit all: the limits of structure-based models in drug discovery. J. Chem. Theory Comput. *9*, 4266–4274.

Schlessinger, A., Geier, E., Fan, H., Irwin, J.J., Shoichet, B.K., Giacomini, K.M., and Sali, A. (2011). Structure-based discovery of prescription drugs that interact with the norepinephrine transporter, NET. Proc. Natl. Acad. Sci. USA *108*, 15810–15815.

Shen, H., Smith, D.E., Yang, T., Huang, Y.G., Schnermann, J.B., and Brosius, F.C. (1999). Localization of PEPT1 and PEPT2 proton-coupled oligopeptide transporter mRNA and protein in rat kidney. Am. J. Physiol. *276*, F658–F665.

Shirts, M.R., Pitera, J.W., Swope, W.C., and Pande, V.S. (2003). Extremely precise free energy calculations of amino acid side chain analogs: comparison of common molecular mechanics force fields for proteins. J. Chem. Phys. *119*, 5740.

Shitara, Y., and Sugiyama, Y. (2002). Inhibition on the transporter-mediated hepatic uptake as a mechanism of drug-drug interaction. J. Pharmacol. Exp. Ther. *30*, 610–616.

Solcan, N., Kwok, J., Fowler, P.W., Cameron, A.D., Drew, D., Iwata, S., and Newstead, S. (2012). Alternating access mechanism in the POT family of oligopeptide transporters. EMBO J. *31*, 1–11.

Sugawara, M., Huang, W., Fei, Y.J., Leibach, F.H., Ganapathy, V., and Ganapathy, M.E. (2000). Transport of valganciclovir, a ganciclovir prodrug, via peptide transporters PEPT1 and PEPT2. J. Pharm. Sci. *89*, 781–789.

Terada, T., Sawada, K., Irie, M., Saito, H., Hashimoto, Y., and Inui, K. (2000). Structural requirements for determining the substrate affinity of peptide transporters PEPT1 and PEPT2. Pflügers Arch. *440*, 679–684.

Trott, O., and Olson, A.J. (2010). Software news and update AutoDock vina: improving the speed and accuracy of docking with a new scoring function, efficient optimization, and multithreading. J. Comput. Chem. *31*, 455–461.

Tsuda, M., Terada, T., Irie, M., Katsura, T., Niida, A., Tomita, K., Fujii, N., and Inui, K.I. (2006). Transport characteristics of a novel peptide transporter 1 substrate, antihypotensive drug midodrine, and its amino acid derivatives. J. Pharmacol. Exp. Ther. *318*, 455–460.

U.S. Department of Health and Human Services, Food and Drug Administration, Center for Drug Evaluation and Research (CDER) (2012). Guidance for industry. Drug interaction studies study design, data analysis, implications for dosing, and labeling recommendations. February 2012.

Vig, B.S., Stouch, T.R., Timoszyk, J.K., Quan, Y., Wall, D., Smith, R.L., and Faria, T.N. (2006). Human PEPT1 pharmacophore distinguishes between dipeptide transport and binding. J. Med. Chem. *49*, 3636–3644.

Wang, W., Wang, J., and Kollman, P.A. (1999). What determines the van der Waals coefficient beta in the LIE (linear interaction energy) method to estimate binding free energies using molecular dynamics simulations? Proteins *34*, 395–402.

Wang, R., Lai, L., and Wang, S. (2002). Further development and validation of empirical scoring functions for structure-based binding affinity prediction. J. Comput. Aided Mol. Des. *16*, 11–26.

Warren, G.L., Andrews, C.W., Capelli, A.M., Clarke, B., LaLonde, J., Lambert, M.H., Lindvall, M., Nevins, N., Semus, S.F., Senger, S., et al. (2006). A critical assessment of docking programs and scoring functions. J. Med. Chem. *49*, 5912–5931.

Yang, W., Bitetti-Putzer, R., and Karplus, M. (2004). Free energy simulations: use of reverse cumulative averaging to determine the equilibrated region and the time required for convergence. J. Chem. Phys. *120*, 2618–2628.

Zhao, Y., Mao, G., Liu, M., Zhang, L., Wang, X., and Zhang, X.C. (2014). Crystal structure of the *E. coli* peptide transporter YbgH. Structure *22*, 1152–1160.

Cell Chemical Biology, Volume *23*

## Supplemental Information

# Accurate Prediction of Ligand Affinities

# for a Proton-Dependent Oligopeptide Transporter

**Firdaus Samsudin, Joanne L. Parker, Mark S.P. Sansom, Simon Newstead, and Philip W. Fowler**

# Accurate prediction of ligand affinities for a peptide transporter – Supplemental Information

Firdaus Samsudin, Joanne L. Parker, Mark S. P. Sansom, Simon Newstead[*] and Philip W. Fowler[†]

Department of Biochemistry, University of Oxford, South Parks Road, OX1 3QU

## Contents

**1 Supplemental Figures** **2**

**2 Supplemental Experimental Procedures** **9**

2.1 Modelling peptide and drug binding . . . . . . . . . . . . . . . . . 9

2.2 Molecular dynamics simulation . . . . . . . . . . . . . . . . . . . . 9

2.3 Binding energy predictions . . . . . . . . . . . . . . . . . . . . . . 10

2.4 Competition transport assays . . . . . . . . . . . . . . . . . . . . . 13

## List of Figures

S1 The 9 poses generated by AutoDock Vina for the dipeptide AF. . . 3

S2 Binding affinity predictions refined by thermodynamic integration . 4

S3 Predicted $\Delta$G values from the LIE method. . . . . . . . . . . . . 5

S4 The N-terminus side chain interacts with a polar cavity while C-terminus side chain occupies a hydrophobic pocket. . . . . . . . 6

S5 Interactions of the lysine side chains of AlaLys and LysAla with residues in the binding site of $\text{PepT}_{\text{St}}$. . . . . . . . . . . . . . . . 7

S6 Binding affinity predictions for a homology model of PepT1. . . . 8

[*]E-mail: simon.newstead@bioch.ox.ac.uk; Corresponding author

[†]E-mail: philip.fowler@bioch.ox.ac.uk; Corresponding author

# 1 Supplemental Figures

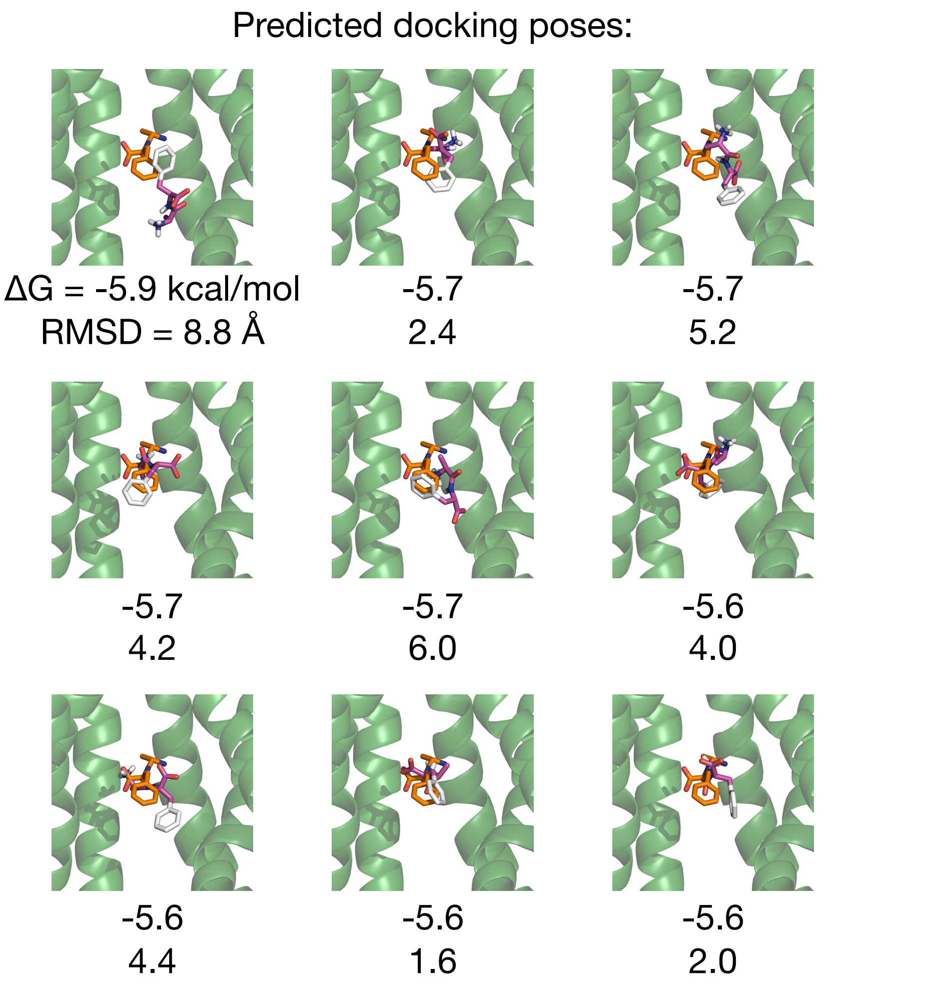


Figure S1: related to Figure 2 - The nine docking poses generated by AutoDock Vina for the dipeptide AlaPhe. These poses are arranged based on $\Delta G$ estimated by a scoring function. The predicted binding orientations (pink) are compared to the crystal structure (orange) and the all-atom RMSD is shown.

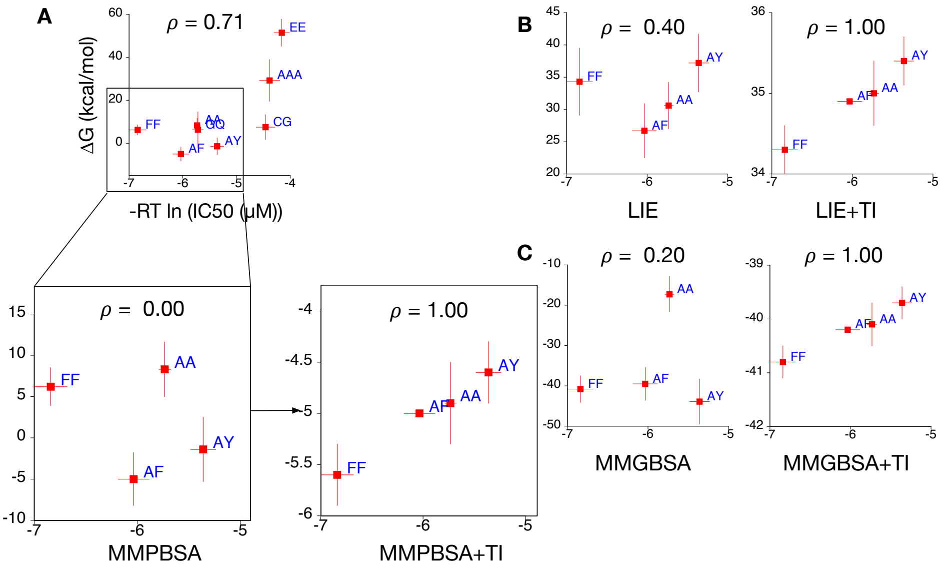


Figure S2: related to Figure 2 - Binding affinity predictions refined by thermodynamic integration. (A) End-point free energy methods like MMPBSA fails to discriminate amongst the good substrates (IC50$\leq$100 $\mu$M) as shown in the enlarged image. TI was employed to calculate $\Delta\Delta$G of these peptides with respect to AlaPhe, and subsequently used to adjust the MMPBSA results. Y-error bars indicate statistical errors from de-correlated and equilibrated $\Delta$G data during MD simulations, while X-error bars indicate the standard deviations from triplicate experiments. The line drawn on each graph represents the least square fit. (B) and (C) illustrate similar refinement steps for LIE and MMGBSA, respectively.

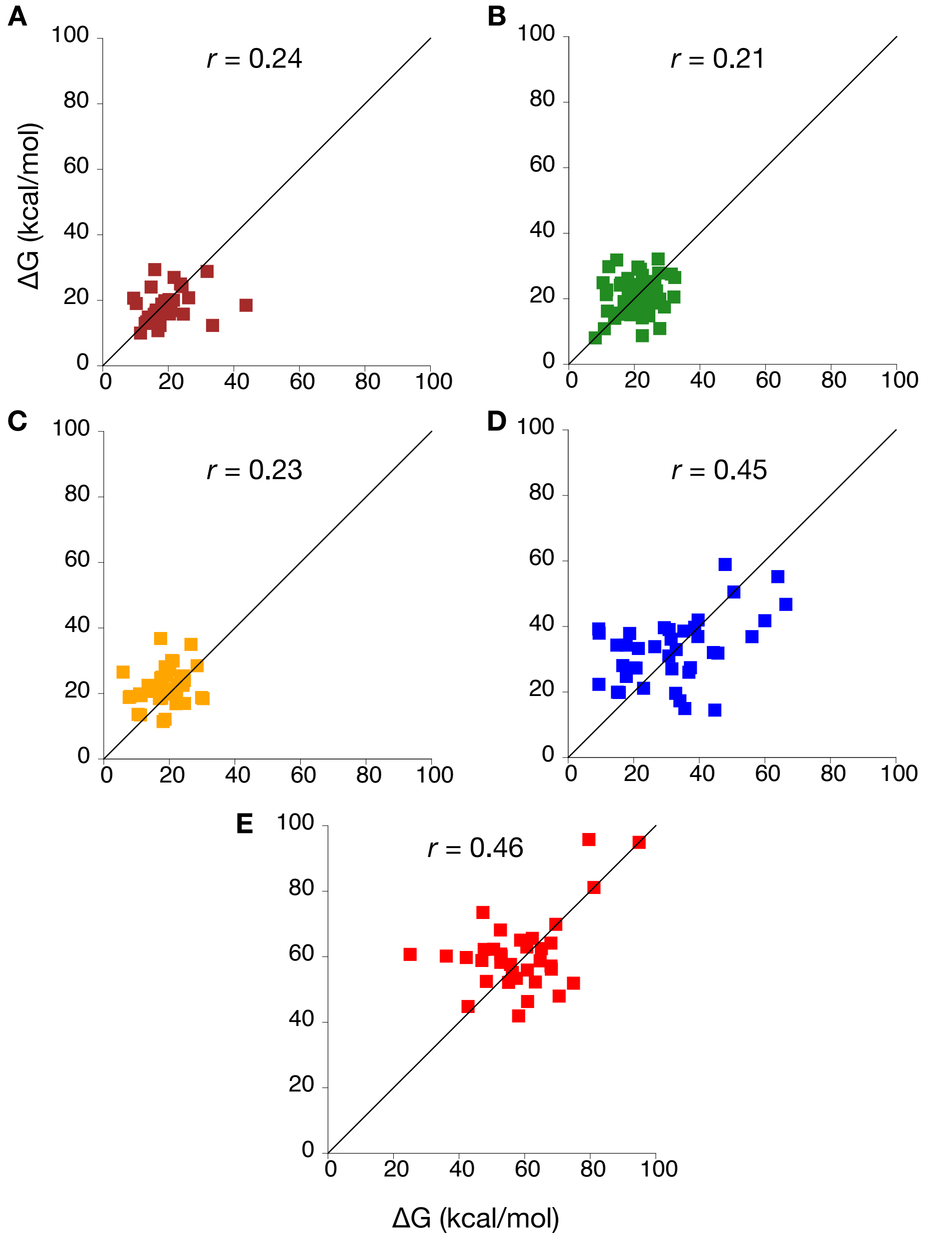


Figure S3: related to Figure 3 - Predicted ΔG values from the LIE method plotted in pairs based on residue combinations, whereby each pair of peptides are made of the same combination of amino acids but in different orders. They are plotted according to the overall chemical properties of the peptides: (A) hydrophobic, (B) polar, (C) other, (D) positive and (E) negative.

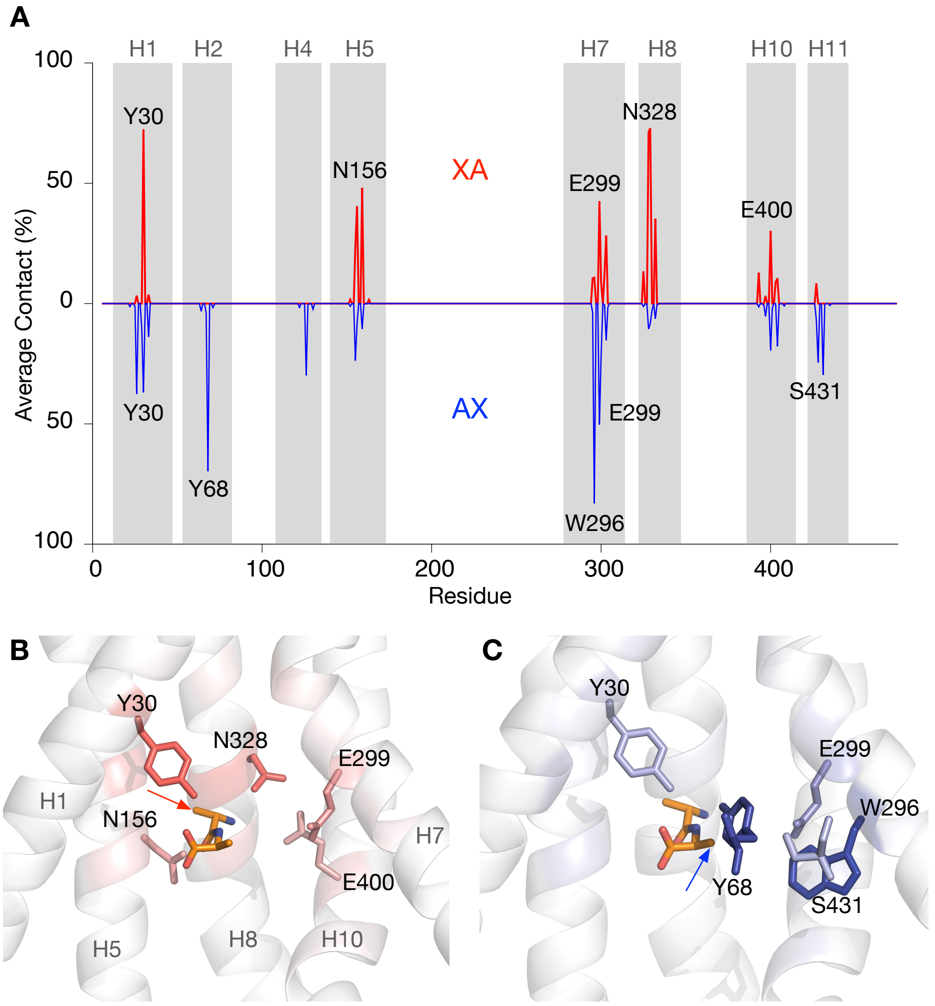


Figure S4: related to Figure 3 - The N-terminus side chain interacts with a polar cavity while C-terminus side chain occupies a hydrophobic pocket. (A) Analysis of contacts made by the side chain of dipeptides Ala-X and X-Ala with $PepT_{St}$ during simulation, whereby X is one of the 20 amino acids. This is averaged over 20 simulations of length 1 ns (one for each amino acid). Residues that make significant contacts are mapped to the binding site ((B) and (C)), while arrows indicate the side chain of interest. The cut-off for contacts is set to 3.5 Å.

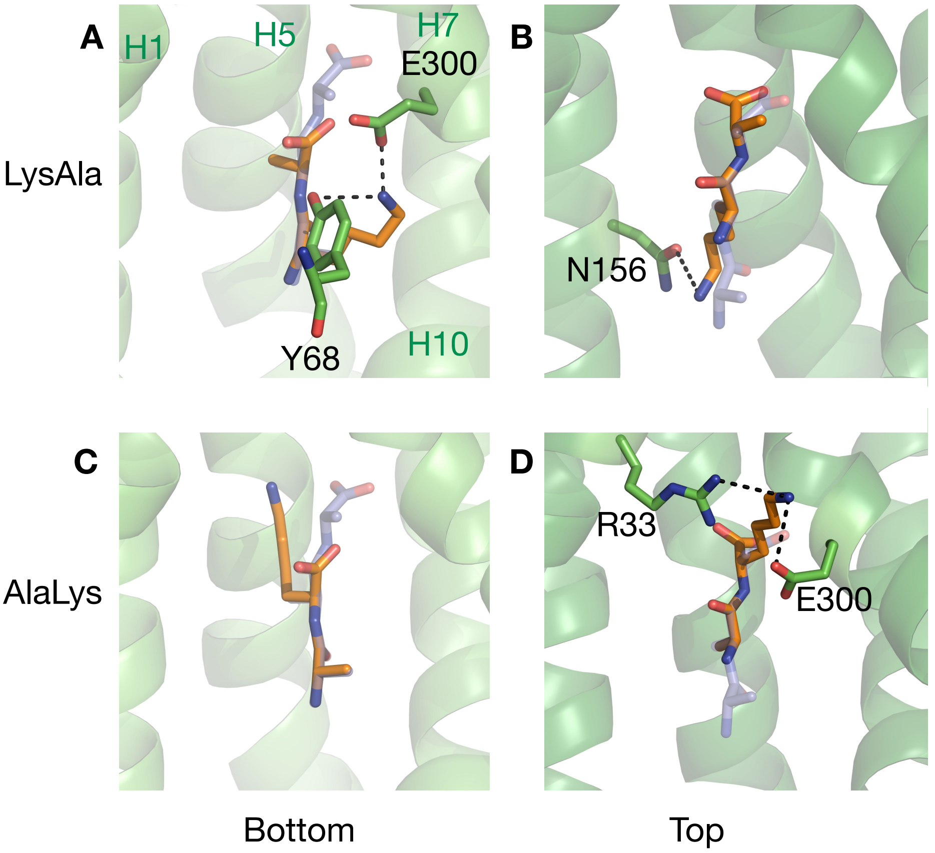


Figure S5: related to Figure 5 - Interactions of the lysine side chains of AlaLys and LysAla (orange stick representations) with residues in the binding site of $PepT_{St}$ (green). Figures show residues that are found within 4 Å of the $\epsilon$-amino group of the side chain and can potentially interact via electrostatic attractions or repulsions. No residues were found in the vicinity of this group for the AlaLys Bottom model. The coordinates of triAla, on which these dipeptides were modelled, are represented in purple.

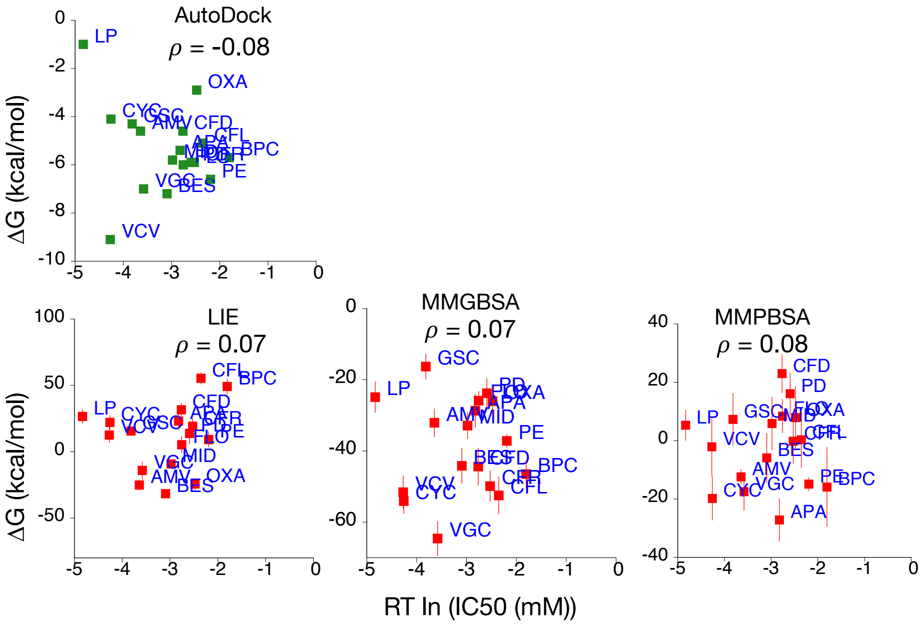


Figure S6: Figure S6, related to Figure 2 and Figure 6 - Extending binding affinity predictions to a homology model of PepT1. Predictions are made using a scoring function (green) and end-point methods (red) for the same peptide test set with additional 14 drug compounds (listed in Experimental procedure). The ability of each method to reproduce experimental ranking is represented as Spearman's correlation coefficient, $\rho$.

## 2 Supplemental Experimental Procedures

### 2.1 Modelling peptide and drug binding

Modelling of peptide substrates were performed using Modeller-9v9 (Sali and Blundell, 1994). The template used for all dipeptides was the crystal complex $PepT_{St}$-AlaPhe (PDB:4D2C) (Lyons et al., 2014). Additionally, the binding of AlaLys and LysAla to $PepT_{St}$ were also modelled based on the structure of $PepT_{St}$-triAla (PDB:4D2D) (Lyons et al., 2014). Only the side chains of the peptide substrate were altered, whereas the structure of the rest of the peptide and protein was kept identical to the template. Models with the highest discreet optimised protein energy (DOPE) scores (Shen et al., 2006) were chosen for MD simulations and $\Delta$G predictions.

Fourteen additional drug compounds were used in the test set of PepT1 homology model: valacyclovir (VCV), cyclacillin (CYC), glycosarcosine (GSC), aminolevulinic acid (AMV), bestatin (BES), valganciclovir (VGC), midodrine (MID), cefadroxil (CFD), floxuridine (FLO), cefaclor (CFR), oxacillin (OXA), aminophenylacetic acid (APA), cefalexin (CFL), and benzylpenicillin (BPC). To model the binding of a drug compound, the structure of the drug was first determined by inspection if it resembles AlaPhe or triAla. Small drugs (AMV, APA, GSC) and prodrugs (VCV, VGC, MID, FLO) are structurally analogous to AlaPhe and were therefore modelled according to the structure of $PepT_{St}$-AlaPhe complex (PDB:4D2C). The $\beta$-lactam antibiotics (CFD, CFL, CFR, CYC, OXA, BPC) and BES are more similar to triAla and hence were modelled based on the structure of $PepT_{St}$-triAla complex (PDB:4D2D).

### 2.2 Molecular dynamics simulation

Transporter proteins and peptide ligands were parametrised using the standard Amber99SB force field (Lindorff-Larsen et al., 2010) while the drug molecules were parametrised using the general Amber force field (GAFF) (Wang et al., 2004). Peptide-bound transporter was embedded in a pre-equilibrated 381-molecule 1-palmitoyl-2-oleoyl-sn-glycero-3-phosphocholine (POPC) model membrane (Dickson et al., 2012) inside a simulation box of dimensions 10 nm $\times$ 13 nm $\times$ 15 nm using the `g_membed` protocol (Wolf et al., 2010). The simulation

box was hydrated by TIP3P water (Jorgensen et al., 1983) and neutralised by 0.15 M $Na^{+}$ and $Cl^{-}$ ions. The steepest-descent protocol was used to minimise the system before being subject to a 1 ns equilibration simulation, during which heavy atoms of the protein and peptide substrates were positionally-restrained. This restraint was removed afterwards for a production MD simulation. Peptide-only simulations underwent the same protocol, albeit without the membrane and in a smaller simulation box (size 3 nm $\times$ 3 nm $\times$ 3 nm). The length of production MD simulations performed for end-point methods is 1 ns and for TI, 5 ns for each $\lambda$.

The temperature within the simulation box was kept at 310 K using the velocity-rescaling thermostat with a time constant of 0.1 ps (Bussi et al., 2007). The pressure was kept at 1 atm by semi-isotropic coupling to a Parrinello-Rahman barostat with a time constant of 1.0 ps and compressibility of $4.5\times10^{-5}$ bar$^{-1}$ (Parrinello, 1981). The lengths of all bonds in the system were constrained using the LINCS algorithm (Hess et al., 1997) and an integration time step of 2 fs was employed by the leap-frog algorithm to solve the Newton's equations of motion. Long-range electrostatic forces were calculated by the particle mesh Ewald (PME) method (Essmann et al., 1995) using a real space cut-off of 12 Å, whereas van der Walls interactions were cutoff at 12 Å. The energy data were written out every 1 ps. All simulations were performed using GROMACS 4.5.4 (Hess et al., 2008) and visualised in PyMOL (Schrödinger) and VMD (Humphrey and Dalke, 1996).

## 2.3 Binding energy predictions

For $\Delta$G predictions using a scoring function, AutoDock Vina (Trott and Olson, 2010) was used to dock each peptide to the binding site. This procedure generates nine binding poses, each with an affinity score. The binding poses were compared to the crystal structures (for AlaPhe and triAla) and models (other peptides and drugs) and the most similar pose according to the RMSD was selected and its binding energy score is taken as the $\Delta$G value. AutoDock Vina was chosen as it implements a scoring function based on X-Score, which has been shown to be the best at ranking ligands (Cheng et al., 2009). No MD simulations were required in this procedure.

To calculate $\Delta$G using the end-point free energy methods, 1 ns MD simulations were performed for each peptide and drug compound. For LIE (Aqvist et al., 1994), two types of simulation were conducted: (i) $PepT_{St}$ bound to a peptide and (ii) the peptide alone in solution. A GROMACS tool, `g_lie`, was used to compute $\Delta$G values along the simulation trajectory based on the energy outputs of both simulations. A default value of 0.18 was used as the non-polar scaling constant, $\alpha$ (Luzhkov and Aqvist, 2001; Osterberg and Aqvist, 2005). For the polar scaling constant, $\beta$, we used the hydroxyl-based parameters as suggested by Hansson et al. (1998), where for charged peptides, $\beta$ = 0.5, for neutral peptides with no hydroxyl group, $\beta$ = 0.43, for neutral peptides with one hydroxyl group, $\beta$ = 0.37 and for neutral peptides with two hydroxyl groups, $\beta$ = 0.33. For MMGBSA and MMPBSA, we follow the single trajectory protocol (Hou et al., 2011), where only the simulation of $PepT_{St}$ bound to a peptide was run and the trajectories of unbound $PepT_{St}$ and free peptide were extracted from it. The `MMPBSA.py` Miller III et al. (2012) program in the AmberTools package, was used to calculate $\Delta$G for both MMGBSA and MMPBSA methods. The salt concentration (`saltcon`) for the generalised Born (GB) calculations and ionic strength (`istrng`) for the Poisson Boltzmann (PB) calculations were both set to 0.15 M.

Alchemical transformation MD simulations were performed to calculate relative free energy of binding $\Delta\Delta$G by the TI method. For our validation step (Figure 2), the crystal structure of $PepT_{St}$-AlaPhe was used for the starting coordinates and the ligand is gradually morphed to either AlaAla, AlaTyr or PhePhe. The dual topology approach was employed, whereby the vanishing and growing atoms were represented separately. For example, during the transformation of AlaPhe to AlaAla, both the phenyl ring and methyl group were attached to the second amino acid residue at the same time. However, branching was done at the $C_\beta$ atom rather than $C_\alpha$ to minimise the number of alchemical transformations. All transformations involved only the non-bonded interactions while bonded interactions were kept the same throughout the simulations (Boresch and Karplus, 1999; Boresch, 2002). Two sets of MD simulations were run using GROMACS 4.5.4 (Hess et al., 2008): (i) the transformation of peptides bound to $PepT_{St}$ (bound simulation) and (ii) the transformation of peptides in solution (unbound). Each transformation was divided into 3 steps: (i) removing the

partial charges of the disappearing chemical groups, (ii) removing the van de Waals interactions of the disappearing groups while adding that of the emerging groups, and (iii) adding the partial charges of the emerging groups. The transformation was done by coupling the non-bonded potential energy terms to a scaling parameter $\lambda$, where at $\lambda$ = 0, the non-bonded terms of AlaPhe were used whilst at $\lambda$ = 1, the non-bonded terms of the final peptide were used. A soft core potential (Beutler et al., 1994) was applied in step (ii) to avoid singularities and instabilities. For each step, we performed 11 independent 5 ns MD simulations at $\lambda$ = 0, 0.1, 0.2, 0.3, 0.4, 0.5, 0.6, 0.7, 0.8, 0.9, 1. The $\partial U/\partial\lambda$ values were computed every 1 ps and extracted using GROMACS `g_energy` tool. These were integrated for all $\lambda$ values using the trapezoid rule along the simulation trajectory to obtain values of $\Delta$G.

For our prediction steps (Figure 3C), a model of PepT$_{\mathrm{St}}$-AlaAla was used for the starting coordinates and the alanine side chain on either the N- or C-terminus was transmuted to phenylalanine, aspartate, glutamate or lysine. For transformations involving a change in the total charge of the peptide substrate, for example from AlaAla to AlaAsp, both the bound and unbound simulations were performed in the same simulation box as per described in Rashid et al. (2013)–while the AlaAla to AlaAsp transformation was applied to PepT$_{\mathrm{St}}$ in the binding site, the reverse transformation (AspAla to AlaAla) was conducted in bulk solution simultaneously to maintain the overall net charge of the system. For predictions with the alternative binding model of AlaLys and LysAla (Figure 5C), the crystal structure of PepT$_{\mathrm{St}}$-triAla was used to model the binding of AlaAla in either the Bottom or the Top models, which is then used as the starting coordinates. Again, either the N- or C-terminus of this AlaAla dipeptide is transformed to lysine.

Equilibration and convergence times for each simulation was estimated based on the reverse cumulative averaging method (Yang et al., 2004). Only de-correlated energy outputs from the equilibrated period of the simulations were used in the calculation of $\Delta$G. For our validation step, the performance of each prediction method was assessed by the Spearman's rank correlation coefficient (Lehmann and D'Abrera, 1998) following the equation below:

$$\rho = 1 - \frac{6\sum(x_i - y_i)^2}{n(n^2-1)}$$

where $x_i$ is the ranking for experimental $IC_{50}$ values, $y_i$ is the ranking for predicted $\Delta$G values and $n$ is the size of the dataset.

## 2.4 Competition transport assays

Proton-driven competition uptake assays were performed as described in Solcan et al. (2012). Proteoliposomes (with 5 $\mu$g $PepT_{St}$) in internal buffer (20 mM Potassium Phosphate, 100 mM Potassium Acetate, 2 mM Magnesium Sulphate, pH 6.5) were diluted into external buffer (120 mM Sodium Phosphate, 2 mM Magnesium Sulphate, pH 6.5) containing 25 $\mu$M 3H labelled AlaAla (reporter substrate) in the presence of increasing peptide concentration (substrate of interest). Reaction was initiated through the addition of 10 $\mu$M valinomycin and terminated after 4 minutes by dilution into 0.1 M Lithium Chloride and collected on nitrocellulose filters prior to scintillation counting. The 3H signal was converted to molar concentrations of peptide using standard curves for each substrate.

## References


Aqvist, J., Medina, C., and Samuelsson, J. (1994). A new method for predicting binding affinity in computer-aided drug design. Protein Eng. *73*, 385–91.

Beutler, T., Mark, A., and van Schaik, R. (1994). Avoiding singularities and numerical instabilities in free energy calculations based on molecular simulations. Chem. Phys. Lett. *222*, 529–539.

Boresch, S. (2002). The Role of Bonded Energy Terms in Free Energy Simulations - Insights from Analytical Results. Mol. Sim. *28*, 13–37.

Boresch, S. and Karplus, M. (1999). The Role of Bonded Terms in Free Energy Simulations: 1. Theoretical Analysis. J. Phys. Chem. A *103*, 103–118.

Bussi, G., Donadio, D., and Parrinello, M. (2007). Canonical sampling through velocity rescaling. J. Chem. Phys. *126*, 014101.

Cheng, T., Li, X., Li, Y., Liu, Z., and Wang, R. (2009). Comparative assessment of scoring functions on a diverse test set. J. Chem. Inf. Model. *49*, 1079–93.

Dickson, C.J., Rosso, L., Betz, R.M., Walker, R.C., and Gould, I.R. (2012). GAFFlipid: a General Amber Force Field for the accurate molecular dynamics simulation of phospholipid. Soft Matter *8*, 9617.

Essmann, U., Perera, L., Berkowitz, M.L., Darden, T., Lee, H., and Pedersen, L.G. (1995). A smooth particle mesh Ewald method. J. Chem. Phys. *103*, 8577.

Hansson, T., Marelius, J., and Aqvist, J. (1998). Ligand binding affinity prediction by linear interaction energy methods. J. Comput. Aided Mol. Des. *12*, 27–35.

Hess, B., Bekker, H., Berendsen, H.J.C., and Fraaije, J.G.E.M. (1997). LINCS: A linear constraint solver for molecular simulations. J. Comp. Chem. *18*, 1463–1472.

Hess, B., Kutzner, C., and Spoel, D.V.D. (2008). GROMACS 4: Algorithms for highly efficient, load-balanced, and scalable molecular simulation. J. Chem. Theory Comput. *4*, 435–447.

Hou, T., Wang, J., Li, Y., and Wang, W. (2011). Assessing the performance of the MM/PBSA and MM/GBSA methods. 1. The accuracy of binding free energy calculations based on molecular dynamics simulations. J. Chem. Inf. Model. *51*, 69–82.

Humphrey, W. and Dalke, A. (1996). VMD: visual molecular dynamics. J. Mol. Graph. *15*, 33–38.

Jorgensen, W.L., Chandrasekhar, J., Madura, J.D., Impey, R.W., and Klein, M.L. (1983). Comparison of simple potential functions for simulating liquid water. J. Chem. Phys. *79*, 926.

Lehmann, E.L. and D'Abrera, H.J.M. (1998). Nonparametrics: Statistical Methods Based on Ranks, Revised (Englewood Cliffs, NJ: Prentice-Hall).

Lindorff-Larsen, K., Piana, S., Palmo, K., Maragakis, P., Klepeis, J.L., Dror, R.O., and Shaw, D.E. (2010). Improved side-chain torsion potentials for the Amber ff99SB protein force field. Proteins *78*, 1950–8.

Luzhkov, V.B. and Aqvist, J. (2001). Mechanisms of tetraethylammonium ion block in the KcsA potassium channel. FEBS Lett. *495*, 191–6.

Lyons, J.A., Parker, J.L., Solcan, N., Brinth, A., Li, D., Shah, S.T., Caffrey, M., and Newstead, S. (2014). Structural basis for polyspecificity in the POT family of proton-coupled oligopeptide transporters. EMBO Rep. 1–8.

Miller III, B., Jr, T.M., Swails, J.M., Homeyer, N., Gohlke, H., and Roitberg, A.E. (2012). MMPBSA.py : An Efficient Program for End-State Free Energy Calculations. J. Chem. Theory Comput. *8*, 3314–3321.

Osterberg, F. and Aqvist, J. (2005). Exploring blocker binding to a homology model of the open hERG K+ channel using docking and molecular dynamics methods. FEBS Lett. *579*, 2939–44.

Parrinello, M. (1981). Polymorphic transitions in single crystals: A new molecular dynamics method. J. Appl. Phys. *52*, 7182.

Rashid, M.H., Heinzelmann, G., Huq, R., Tajhya, R.B., Chang, S.C., Chhabra, S., Pennington, M.W., Beeton, C., Norton, R.S., and Kuyucak, S. (2013). A potent and selective peptide blocker of the Kv1.3 channel: Prediction from free-energy simulations and experimental confirmation. PLoS One *8*.

Sali, A. and Blundell, T. (1994). Comparative protein modelling by satisfaction of spatial restraints. J. Mol. Biol. *234*, 779–815.

Shen, M., Devos, D., Melo, F., and Sali, A. (2006). A composite score for predicting errors in protein structure models. Protein Sci. *15*, 1653–1666.

Solcan, N., Kwok, J., Fowler, P.W., Cameron, A.D., Drew, D., Iwata, S., and Newstead, S. (2012). Alternating access mechanism in the POT family of oligopeptide transporters. EMBO J. 1–11.

Trott, O. and Olson, A.J. (2010). Software News and Update AutoDock Vina : Improving the Speed and Accuracy of Docking with a New Scoring Function, Efficient Optimization, and Multithreading. J. Comput. Chem. *31*, 455–461.

Wang, J., Wolf, R.M., Caldwell, J.W., Kollman, P., and Case, D. (2004). Development and testing of a general amber force field. J. Comp. Chem. *25*, 1157–74.

Wolf, M.G., Hoefling, M., Aponte-santamaría, C., Grubmüller, H., and Groenhof, G. (2010). g_membed : Efficient Insertion of a Membrane Protein into an Equilibrated Lipid Bilayer with Minimal Perturbation. J. Comp. Chem. *31*, 2169–2174.

Yang, W., Bitetti-Putzer, R., and Karplus, M. (2004). Free energy simulations: use of reverse cumulative averaging to determine the equilibrated region and the time required for convergence. J. Chem. Phys. *120*, 2618–28.
